# A new oviraptorosaur (Dinosauria: Theropoda) from the end-Maastrichtian Hell Creek Formation of North America

**DOI:** 10.1371/journal.pone.0294901

**Published:** 2024-01-24

**Authors:** Kyle L. Atkins-Weltman, D. Jade Simon, Holly N. Woodward, Gregory F. Funston, Eric Snively

**Affiliations:** 1 Oklahoma State University, Tahlequah, OK, United States of America; 2 Department of Ecology and Evolutionary Biology, University of Toronto, Toronto, Ontario, Canada; 3 Royal Ontario Museum, Toronto, Ontario, Canada; Universidade de Sao Paulo, BRAZIL

## Abstract

Caenagnathidae is a clade of derived, Late Cretaceous oviraptorosaurian theropods from Asia and North America. Because their remains are rare and often fragmentary, caenagnathid diversity is poorly understood. *Anzu wyliei* is the only caenagnathid species currently described from the late Maastrichtian Hell Creek Formation of the USA and is also among the largest and most completely preserved North American caenagnathids. Smaller, less complete caenagnathid material has long been known from the Hell Creek Formation, but it is unclear whether these are juvenile representatives of *Anzu* or if they represent distinct, unnamed taxa. Here, we describe a relatively small caenagnathid hindlimb from the Hell Creek Formation, and conduct osteohistological analysis to assess its maturity. Histological data and morphological differences from *Anzu wyliei* and other caenagnathids allow us to conclude that this specimen represents a new species of caenagnathid from the Hell Creek Formation, with a smaller adult body size than *Anzu*. This new taxon is also distinct from other small caenagnathid material previously described from the area, potentially indicating the coexistence of three distinct caenagnathid species in the Hell Creek Formation. These results show that caenagnathid diversity in the Hell Creek ecosystem has been underestimated.

## Introduction

Oviraptorosauria was a clade of maniraptoran theropods characterized by a foreshortened rostrum, often bearing an edentulous beak, and highly pneumatic skeletons, a combination that easily distinguishes them from other theropod groups [1]. Oviraptorosaurs are known from well-preserved fossils, primarily from Asia, that have yielded insight into the biology, morphology, diversity, and evolutionary history of this peculiar group of theropods [1–19]. The earliest certain oviraptorosaurs are known from the Barremian stage of the Early Cretaceous, between 125–129 million years ago [20,21], although links have recently been drawn with the unusual scansoriopterygids [13] and *Ornitholestes* [13], potentially reconciling a gap that extends into the Jurassic between their presumed divergence from other coelurosaurs and their earliest appearances. The Late Cretaceous record of oviraptorosaurs is excellent, and it is clear that they persisted until the K-Pg extinction event in Laurasia [12].

Oviraptorosaurs ranged from chicken- or turkey-sized [3,4], to species which likely weighed more than a tonne [5]. Based on their cranial morphology, this diverse clade contained both omnivorous and herbivorous members [12,21–23]. Although the earliest members of this group retained teeth [3], they were entirely lost in Late Cretaceous forms. Well-preserved fossils preserve the feathery integument [3,6–8], quill knobs on the ulna [9], and specialized vertebrae that form a pygostyle structure [10–14], showing that oviraptorosaurs had complex, feathery integument. Other exquisite specimens have shown that these dinosaurs were social [2,24,25], actively brooded over their nests [15,19,26–31], employed a reproductive strategy intermediate between those used by crocodilians and extant birds [32], and had avian-like brain organization [33,34] (see [20] for a more detailed examination of this topic).

The excellent oviraptorosaur record is overwhelmingly known from the Late Cretaceous of Asia, and thus the true diversity across their entire range is incomplete. Throughout the past two decades, multiple new species have been discovered in North America, leading to a better understanding of their morphology and interrelationships [12,35–42]. All North American oviraptorosaurs fall within the monophyletic family Caenagnathidae [8,12,35,38,42–45], which was also present but less diverse in Asia [5,39] during the Late Cretaceous. Caenagnathids can be easily characterized by their slender manus, gracile, long hindlimbs, and distinctive mandibles with complexly-textured edentulous beaks in place of the toothed jaws seen in other theropods [35,40]. North American caenagnathid fossils are often found as isolated elements or are poorly preserved, and thus they have historically been misidentified as members of other clades [46], or subject to taxonomic issues where isolated specimens cannot be associated due to a lack of overlapping material [see [41] for a review]. However, a number of discoveries have led to an improved resolution of caenagnathid relationships and their links to other members of Oviraptorosauria [12,35–37,45,47].

Nevertheless, our understanding of caenagnathid diversity remains plagued by the relative incompleteness of most specimens, with most taxa known from very sparse material [35,37,41,43–45]. Thus, caenagnathid taxonomy and variation is debated and unstable. Two major advances in recent years have improved the situation. The first was the discoveries of three exceptionally complete caenagnathid skeletons and their description as *Anzu wyliei* [12], which remains one of the most completely known caenagnathids. This discovery drastically improved the phylogenetic representation of caenagnathids, and helped clarify different aspects of caenagnathid evolution [41,47–49]. *Anzu wyliei* specimens further elucidated the unusual skulls and skeletal proportions of caenagnathids, and expanded the skeletal representation of large-bodied North American caenagnathids, which had previously only been represented by eggshell [50]. The second major advance is the leverage of histological data by multiple research groups, to assess the relative maturity of specimens, so that ontogeny can be addressed in taxonomic hypotheses [51,52]. Together, these two advances have resulted in improved taxonomic frameworks with falsifiable hypotheses and provide a robust strategy for unravelling the broader diversity of caenagnathids.

However, some discrepancies in caenagnathid diversity remain. Differences in species richness between Asia and North America were recently addressed [53], but differences within North American ecosystems are less clear [41,44]. Whereas caenagnathids were highly diverse in the Campanian [37,41,45,47], they appear to be less diverse in the Maastrichtian, with *Anzu wyliei* as the only named taxon [12]. Nonetheless, some small caenagnathid material from the Hell Creek Formation exists, and has previously been referred to what is now *Citipes elegans* (= *Elmisaurus elegans*) [32,34,38,42], although this has not been reevaluated since that species was transferred to a new genus. Currently unpublished descriptive and histological work [52] suggests that multiple taxa may exist in the Hell Creek Formation, but the ontogenetic status of the smaller material is unknown, and the caenagnathid diversity of this formation requires reevaluation.

Here we describe the morphology and osteohistology of a new, partial caenagnathid hindlimb intermediate in size between known specimens from the Hell Creek Formation. Building on recent work [46,51,52], we anticipated that this specimen might provide an opportunity to assess growth across ontogeny in *Anzu wyliei*. Surprisingly, our morphological and histological results indicate that the specimen does not represent a juvenile *Anzu wyliei*, but a morphologically-distinct adult individual approaching asymptotic body size, representing a new taxon. This prompted a reevaluation of the diversity of caenagnathids in the Hell Creek Formation, which has ramifications for the diversity of oviraptorosaurs prior to the end-Cretaceous extinction.

## Materials and methods

**Fig 1:**
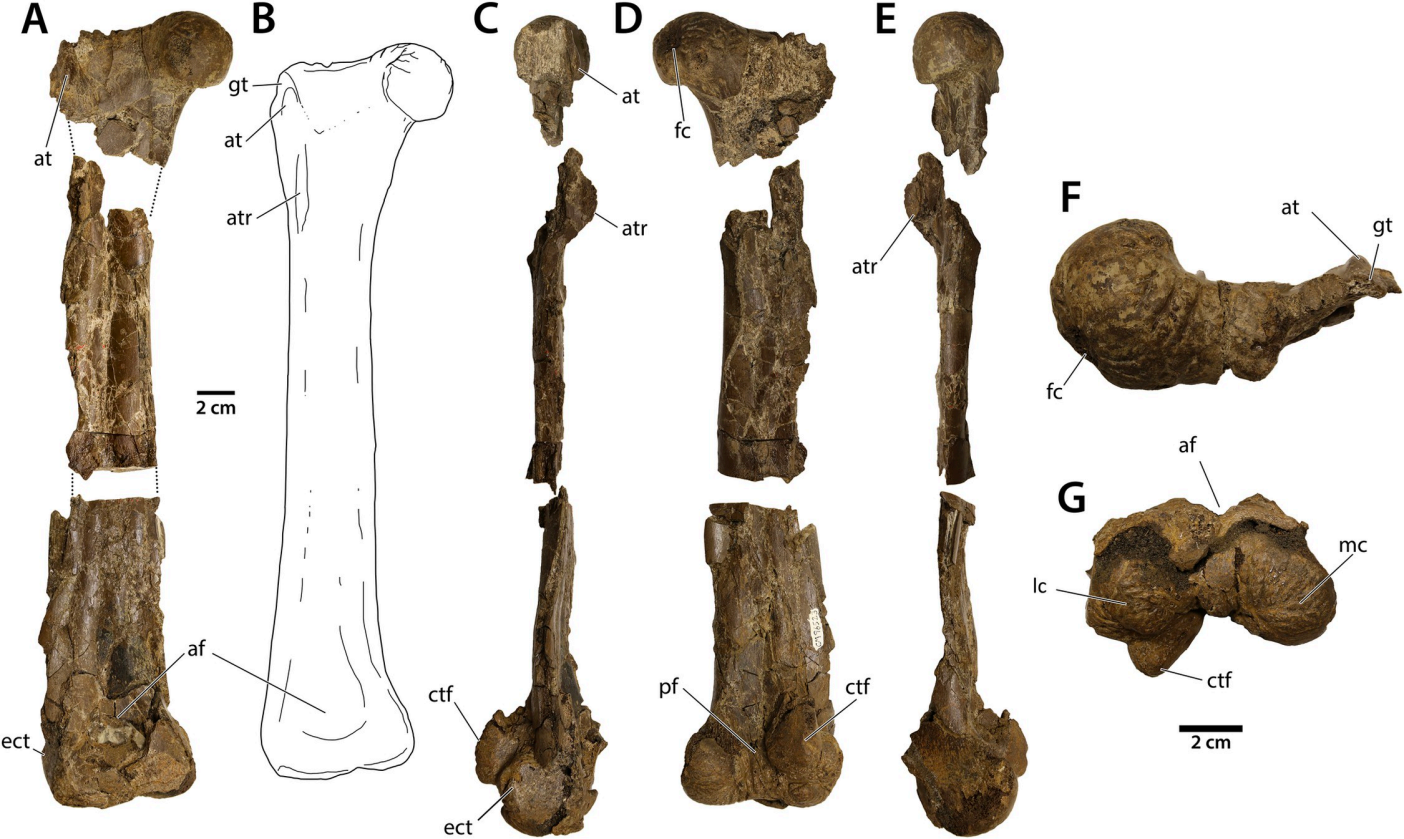
Left femur of CM 96523. A) Anterior view, showing connections between the three fragments as presently preserved. The distal two fragments were initially united before palaeohistological sampling. B) interpretive illustration of femur; C) lateral view, D) posterior view, E) medial view, F) proximal view, and G) distal view. Abbreviations: af, adductor fossa; at, anterior trochanter; atr, accessory trochanteric ridge; ctf, crista tibiofibularis; ect, ectocondylar tuber; fc, fovea capitis, gt, greater trochanter; mc, medial condyle; pf, popliteal fossa; tc, tibial condyle. Scale bar = 2 cm.

CM 96523 comprises a partial right hindlimb including femur, tibia, astragalocalcaneum, and metatarsals III and IV. All bones were prepared prior to acquisition. The femur was received with the femoral head attached incorrectly, and this error was rectified by preparators at the Carnegie Museum of Natural History (CM), Pittsburgh, Pennsylvania, United States of America. Likewise, some remaining matrix was removed from the tibia. The femur and tibia were measured using ImageJ, while the metatarsals were measured using a flexible tape measure (Table 1). Measurements were taken from the proximal end to the distal end of the element, to the nearest millimeter. Other measurements such as proximodistal width at the proximal and distal ends were taken digitally using prepared figures (specifically Fig 1A and 1B in case of the femur) and ImageJ. The cnemial crest of the tibia was distorted, and a tibia proximal width measurement would be unreliable and thus was not taken.

**Table 1:** Measured length of preserved elements of *Eoneophron infernalis*. All measurements are in mm.

Bone	Femur	Tibia +Astragalocalcaneum	Mt III	Mt IV
**Length**	390	499	247	233
**Circumference [est]**	105.8	106.8	-	64.2
**Proximal width**	100	-	15	21
**Distal width**	78	70	21	15

### Paleontological ethics statements

CM 96523 is permanently reposited in the collections of the Section of Vertebrate Paleontology at CM, where the locality information for this specimen is on file. No permits were required for the described study, which complied with all relevant regulations. All specimens were collected from privately-owned land in the United States of America with the written consent of the respective landowners and were purchased by the primary author for donation to the Carnegie Museum.

### Institutional abbreviations

**BHM**, Black Hills Institute of Geological Research, Hill City, South Dakota, United States of America; **CM**, Carnegie Museum of Natural History, Pittsburgh, Pennsylvania, United States of America; **FMNH**, Field Museum of Natural History, Chicago, Illinois, United States of America; **MPC**, Mongolian Paleontological Center, Ulaanbaatar, Mongolia; **MRF**, Marmarth Research Foundation, Marmarth, North Dakota, United States of America; **OMNH**, Sam Noble Oklahoma Museum of Natural History, Norman, Oklahoma, United States of America; **ROM**, Royal Ontario Museum, Toronto, Ontario, Canada; **TMP**, Royal Tyrrell Museum of Palaeontology, Drumheller, Alberta, Canada; **UALVP**, University of Alberta Laboratory for Vertebrate Palaeontology, Edmonton, Alberta, Canada.

### Nomenclatural acts

The electronic edition of this article conforms to the requirements of the amended International Code of Zoological Nomenclature, and hence the new names contained herein are available under that Code from the electronic edition of this article. This published work and the nomenclatural acts it contains have been registered in ZooBank, the online registration system for the ICZN. The ZooBank LSIDs (Life Science Identifiers) can be resolved and the associated information viewed through any standard web browser by appending the LSID to the prefix “http://zoobank.org/”. The LSID for this publication is: urn:lsid:zoobank.org:pub: XXXXXXX. The electronic edition of this work was published in a journal with an ISSN, and has been archived and is available from the following digital repositories: LOCKSS [author to insert any additional repositories].

### Histological sectioning

The region containing the minimum circumference within the mid-diaphyses of the femur, tibia, and metatarsal IV were selected for removal and thin section processing, following procedures outlined in [54]. Lines of minimum circumference were marked with red wax pencil. Prior to sample removal, each bone was photographed in anterior, posterior, lateral, and medial orientations; bone shape was outlined on paper; and each bone was 3D laser scanned using a NextEngine Ultra HD scanner. Finally, plaster cradles were made for each specimen.

Transverse samples that included the minimum diaphysis circumference were removed using a wet tile saw fitted with a continuous rim diamond blade. On either side of the sample removed, the specimen suffered a small amount of kerf loss from the blade. A small break was forced upon nearly completing each transverse cut through the shafts, to ensure one area on each side of the sample matched up to the rest of the diaphysis, to preserve the original length of the bone once the cast was produced. Each sample removed was then molded using Douglas and Sturgess 2-part Silputty, and cast using Smooth-On Smooth-Cast 321 two-part resin. Casts were primed and painted to resemble the appearance of the removed samples. The cast replicas were not restored to the bone shafts, but placed in the specimen cradles where the original pieces were removed. The portion of the cast representing the forced break in the samples contacted with the remainder of the specimen, ensuring no loss in length in each case with inclusion of the replica.

The sample removed was indicated on each drawn specimen outline, and the samples were then embedded in Silmar two-part polyester resin (Sil95BA-41). After the resin cured, the samples were processed using a Buehler Isomet 1000 precision saw, with a diamond wafering blade, to obtain two, 2 millimetre-thick transverse wafers on either side of the minimum diaphysis. For each sample one surface of each wafer was then polished on a Buehler Ecomet 4 variable speed polisher/grinder with 600 grit, then 800 grit silicon carbide paper to remove marks left by the wafering blade. The polished side of the wafers were then glued to frosted plastic slides using cyanoacrylate glue (Starbond, medium viscosity) and left to cure for 48 hours. Each slide was then polished on the Ecomet 4 using silicon carbide papers of progressively finer grit (60 to 1200), and hand polished using a 1 micron cloth with 5 micron slurry and finally with 1 micron aluminum oxide gel.

Each completed thin section was analyzed using a Nikon Eclipse polarizing microscope under plane, circular, and full wave-plate polarization. Whole thin section photomontages were produced using an ASI motorized stage and Nikon DS-Ri2 camera affixed to the microscope at 50X total magnification, and with the software package Nikon Elements: Documentation. The femur and tibia diaphyses suffered post-burial crushing, so the resulting digital images of the femur and tibia were digitally restored to their original shape using Adobe Photoshop CC. Digital Line of Arrested Growth (LAG) and surface tracing was also performed in Photoshop for all three bones (which included the digitally reconstructed femur and tibia). Each LAG, where visible, was digitally traced on a Photoshop layer using a blue line, and a red line was used to indicate a region where LAG tracings and surface tracings had to be estimated due to cortical drift, crushing, or missing cortex. Diaphyseal circumferences were quantified using Fiji [55].

### Mass estimation

In order to estimate the body mass of CM 96523, it was necessary to first estimate the circumference of the femur, accounting for the taphonomic distortion. This was done by digitally retrodeforming the cross section of the femur based on femoral cross sections generated by computed tomography scans of other oviraptorosaurs like *Oksoko* [2] and *Apatoraptor* [47]. This estimate was then plotted against regressions of body mass versus stylopodial circumference made by Campione and others [56] to obtain a range of plausible body masses for this specimen. We did the same for *Anzu*, using the femoral circumference of CM 78000 provided by Lamanna et al. [12] to compare their masses using the same method of estimation.

### Phylogenetic methods

To determine the phylogenetic position of the specimen, it was included as an operational taxonomic unit in the oviraptorosaur phylogenetic matrix of Funston et al. 2020 [2]. CM 96523 could be scored for 23 characters (9.4%) in the dataset, which comprised 42 taxa and 246 characters. A cladistic analysis was run in TNT using 10,000 replications of Wagner Trees and a subsequent round of Tree Bisection Reconnection to find the shortest trees. Time calibration of the phylogeny was done using the *strap* package in R and the age of the Hell Creek Formation [12] for CM 96523.

## Results

### Systematic paleontology

Theropoda Marsh 1878 [57]

Oviraptorosauria Barsbold 1976 [58]

Caenagnathidae Sternberg 1940 [59]

*Eoneophron* gen. nov.

[LSID from ZooBank]

*Eoneophron infernalis* sp. nov.

[LSID from ZooBank]

#### Holotype

CM 96523, partial hindlimb including right femur, right tibia and astragalocalcaneum, a right metatarsal III, and a right metatarsal IV.

#### Etymology

Genus name derived from the Ancient Greek “eo”–meaning “dawn,” and from the genus name of the Egyptian vulture, *Neophron*, sometimes referred to as the “pharaoh’s chicken.” The species name derives from Latin for Hell, in reference to the Hell Creek Formation. Together the taxon name equates to “Pharaoh’s dawn chicken from Hell.”

#### Locality and horizon

Collected from exposures of the upper Maastrichtian [60] Hell Creek Formation, Meade County, South Dakota, United States of America. The specimen was prepared prior to acquisition, but matrix remains adhered to some areas, such as the proximal portion of the tibia and anterodistal region of the femur. These sediments resemble those of typical floodplain deposits such as siltstones with organic material as seen elsewhere in the Hell Creek Formation (KLAW, pers. obs.).

#### Diagnosis

Caenagnathid oviraptorosaur diagnosed by the following combination of traits (autapomorphies are denoted with an asterisk): femoral head directed dorsomedially, rather than perpendicular to the shaft; astragalocalcaneum fused to tibia*, metatarsal III with well-developed posterior cruciate ridges continuous with the distal condyle; distal condyle of metatarsal III transversely wider than anteroposteriorly deep, with medial portion of condyle larger than lateral portion; proximal end of metatarsals III and IV project posteriorly to form proximal protuberance; distal tarsal IV coossified with the proximal end of metatarsal IV at maturity; shaft of metatarsal IV with well-developed oblique longitudinal ridge on anterior surface extending along the distal three-quarters of the shaft*.

#### Taxonomic comments

In order to assess the taxonomic identification of the specimen, we followed the approach used by Cullen et al. 2020 on specimen ROM VP 65884, and scored *Eoneophron infernalis* for the data matrix of Funston et al. (2020). We then compared character states and morphological characters to other oviraptorosaurs, particularly focusing on the coeval *Anzu wyliei* and other Late Cretaceous caenagnathids of North America. *E*. *infernalis* exhibits several characters of the metatarsus noted to be diagnostic of the clade Caenagnathidae [46]. Specifically, the semi-arctometatarsalian relationship of the proximal ends of metatarsals III and IV, the anteroposteriorly flattened metatarsal III, the prominent and deep concavity on the posterior surface of the tarsometatarsus, and the cruciate ridges on the posterior side of metatarsal III are diagnostic of caenagnathids [2,12,41,42,45,53]. While the specimen’s incompleteness makes it difficult to place within Caenagnathidae with any great precision, it does share some affinities with *Citipes elegans* and *Elmisaurus rarus* in the pronounced development of the cruciate ridges on metatarsal III, the development of a proximal posterior protuberance on metatarsals III and IV, and the fusion of distal tarsal IV to metatarsal IV. This affinity is supported by its position recovered in the phylogenetic analysis.

### Description and comparisons

CM 96523 consists of the right femur, right tibia and astragalocalcaneum, right metatarsal III, and right metatarsal IV. Both the femur and tibia are dorsoventrally crushed along their shafts, however, proximal and distal ends of these two bones remain relatively unaltered. The bone surface is fragmented, likely due to pressure undergone during the fossilization process. In contrast, the two preserved metatarsals show no signs of crushing, and are entirely unbroken aside from a few small cracks. All bones have laminated, smooth surface textures, although there are clear changes in surface texture on articular surfaces and areas of muscle or ligament attachment.

#### Femur

The femur of *Eoneophron infernalis* is shorter than any complete femora assigned to *Anzu wyliei* [12] (Table 1), but is similar in length to known specimens of cf. *Caenagnathus collinsi* and *Chirostenotes pergracilis* [41]. The distal and proximal ends are preserved well enough to determine several anatomical features (Fig 1), but the shaft is crushed. The femoral head has a series of distinct furrows, indicating a prominent cartilaginous epiphysis; these furrows are not quite as prominent in CM 78000 or CM 78001 (*Anzu wyliei*). The head of the femur is dorsomedially directed, so that it meets the long axis of the shaft at an obtuse angle of approximately 100°, which is similar to TMP 1986.036.0323 (cf. *Caenagnathus collinsi*) [42] and some oviraptorids like *Oksoko avarsan* [2] and *Rinchenia mongoliensis* [61]. In contrast, in other caenagnathids like *Chirostenotes pergracilis* [35], *Elmisaurus rarus* [62], *Nomingia gobiensis* [11], and *Anzu wyliei*, the femoral head is directed medially and meets the shaft at roughly a 90° angle [12]. As in *Anzu* and other caenagnathids, there is a shallow groove separating the greater trochanter and femoral head, and it appears that the anterior and greater trochanters are equally tall and form a trochanteric crest, although the anterior trochanter is damaged and its morphology is difficult to discern. There is a small accessory trochanteric crest distal to the anterior trochanter, but its size is exaggerated by the crushing of the bone. This crest is more poorly developed than in *Chirostenotes pergracilis* [35], *Elmisaurus rarus* [62], or *Nomingia gobiensis* [11].

There is no fourth trochanter in *Eoneophron infernalis*, as is the case in most oviraptorosaurs. The adductor fossa and the associated anteromedial crest on the distal femur are well-developed, as seen in *Anzu*, but not quite to the same degree. The distal condyles of CM 96523 are relatively smaller than those of *Anzu*, but this may be allometrically related to the smaller size of the element. There is a small ectocondylar tuber on the lateral side of the fibular condyle; this is comparable in size to those of other caenagnathids, but distinctly smaller than those of avimimids [24] or oviraptorids [2,61]. As in all oviraptorosaurs, the lateral condyle projects distally slightly beyond the medial condyle and has a well-developed notch separating the condyles of the crista tibiofibularis.

#### Tibia and astragalocalcaneum

The tibia is long and slender: the ratio of the distal condyle width to the proximodistal length is less than 0.20. The tibia is approximately 128% of the length of the femur, differing slightly from the proportions seen in CM 78000 and CM 78001, where the tibia is between 1.25 and 1.17 times longer than the femur respectively [12]. The cnemial crest of the tibia protrudes far anteriorly, and has a rounded anterior outline in medial or lateral view. It is restricted to the proximal part of the shaft, not extending more than 15% the length of the tibiotarsus, on par with those of other caenagnathids [35,47,62]. The apex of the cnemial crest is at the ventral edge of its extent, similar to other oviraptorosaurs but differing from other families of theropods like ornithomimids, alvarezsaurs, and dromaeosaurs [47]. The edge of the cnemial crest is tranversely thickened into a knob, which is widest dorsally and tapers ventrally. The cnemial crest is separated from the fibular condyle by a deep incisura tibialis. The fibular condyle is relatively large and bulbous, and it appears to be separated from the posterior edge of the proximal end by a shallow notch. The fibular crest is ridge-like and grades into the lateral edge of the shaft at either end. It ends 33% of the length of the tibia from the proximal end, as in *Chirostenotes pergracilis* [35]. A shallow concavity on the distal end of the shaft marks the area where the ascending process of the astragalus would have attached; only the base of the ascending process is preserved. From this impression, it appears that the ascending process of the astragalus was taller than its width at the base, which is the case in all oviraptorosaurs [1]. The postfibular flange is less well developed than that of *Elmisaurus rarus* [62], TMP 1994.012.0880 (cf. *Citipes elegans*) or RSM P2600.1 (Caenagnathidae indet. from the Frenchman Formation of Saskatchewan, Canada) [39].

**Fig 2:**
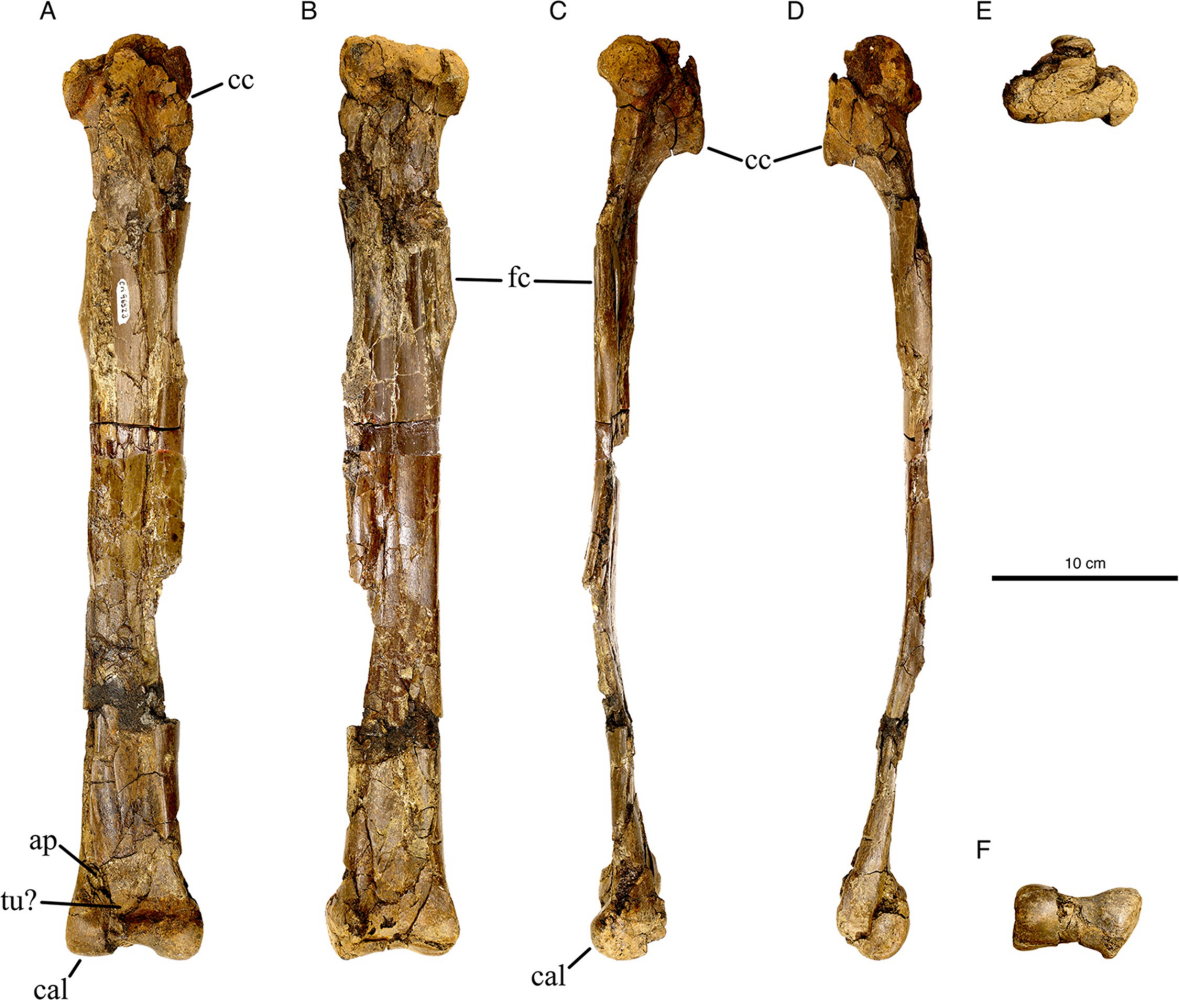
Right tibia and astragalocalcaneum of *Eoneophron infernalis*. In A) Anterior, B) Posterior, C) Lateral, D) Medial, E) Proximal, and F) Distal views. Abbreviations: ap, ascending process; cal, calcaneum; cc, cnemial crest; fc, fibular crest; tu?, tubercle? Scale bar = 10cm.

**Fig 3:**
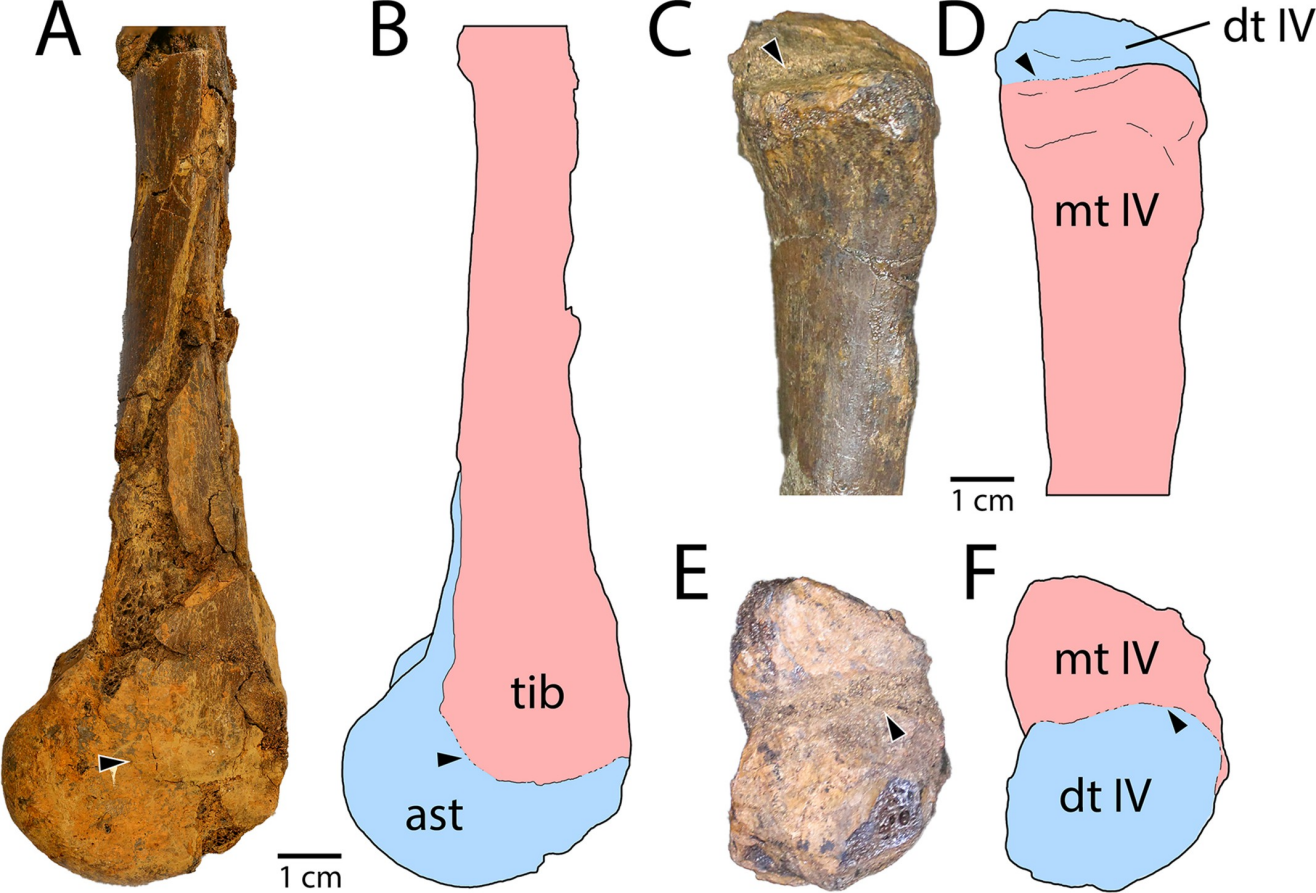
Coossification of hindlimb elements in *Eoneophron infernalis*. A) Tibiotarsus in medial view, showing coossification of the astragalocalcaneum and tibia (arrow). B) Interpretive illustration of (A). C) Distal tarsal IV and proximal end of Metatarsal IV in anterior view, showing fusion of the distal tarsal to the proximal metatarsal (arrow). D) interpretive illustration of (C). E) Distal tarsal IV and Metatarsal IV in proximal view, showing fusion of the elements (arrow). F) Interpretive illustration of (E). Abbreviations: ast, astragalus; dt IV, distal tarsal IV; mt IV, Metatarsal IV; tib, tibia.

The astragalus and calcaneum are fused into a single element without a suture (Fig 2), as is seen in *Anzu wyliei* [12] but not all other caenagnathids, including Maastrichtian examples (RSM P2600.1) [39,53,62]. Surprisingly, the astragalocalcaneum itself appears to be coossified with the end of the tibia (Fig 3), a trait that is so far unknown in caenagnathids, but has been described in *Avimimus* [24] and observed in an undescribed partial tibiotarsus, possibly also from a caenagnathid, from the Dinosaur Park Formation of Alberta (TMP 1985.065.0001; GFF pers. obs.). The latter specimen (TMP 1985.065.0001) is catalogued as an ornithomimid, but shares several features with caenagnathids, including a semi-circular cross-section of the shaft and the absence of a groove or ridge for the fibula (which sits lateral to the tibia throughout its length in oviraptorosaurs, as opposed to other theropods including ornithomimids). The condyles of the astragalocalcaneum in *Eoneophron infernalis* are uneven in size, and the medial condyle is larger and extends further distally. In RSM P2600.1, the condyles are more similar in size and extend equally far distally [39]. The medial condyle is fused to the medial malleolus of the tibia, whereas a suture can still be discerned between the postfibular flange and the lateral edge of the tibia. Due to surface crushing, it is difficult to determine with certainty whether or not there is a tubercle on the astragalocalcaneum, but if so, it is not nearly as well developed as is in *Anzu* [12].

#### Metatarsals

The two metatarsals found in *Eoneophron infernalis* cannot be compared to the type series of *Anzu wyliei*, as no weight-bearing metatarsals were preserved in the latter specimens. While a partial set of weight-bearing metatarsals were associated with ROM VP 65884, referred by Cullen et al. to cf. *Anzu wyliei* [46], none of the three were complete and each was missing the proximal ends and distal condyles. However, more complete metatarsals III and IV, probably pertaining to *Anzu wyliei*, were described by Tsujimura et al. (2021), and metatarsals are also known for the indeterminate Maastrichtian caenagnathids from the Frenchman Formation (RSM P2600.1) [39]. Both provide valuable comparisons for CM 96523 (*Eoneophron infernalis*) [63].

**Fig 4:**
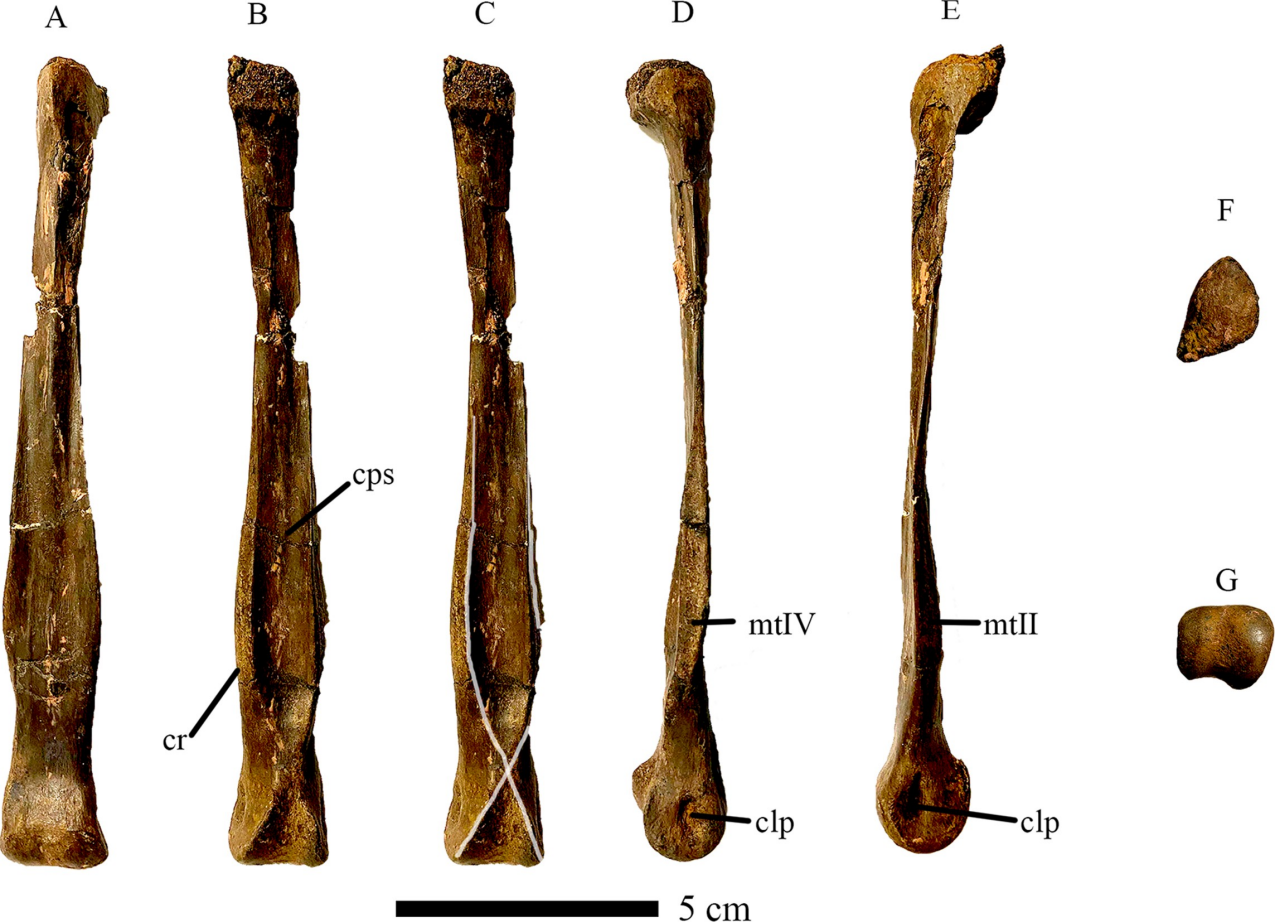
Right Metatarsal III of CM 96523. In A) Anterior, B) Posterior, C) Posterior (cruciate ridges highlighted), D) Lateral, E) Medial, F) Proximal, and G) Distal views. Cruciate ridges highlighted in white where visible. Abbreviations: clp, collateral ligament pit; cps, concave posterior surface; cr, cruciate ridges; mtII, articular surface for Metatarsal II; mt IV, articular surface for Metatarsal IV. Scale bar = 5cm.

**Fig 5:**
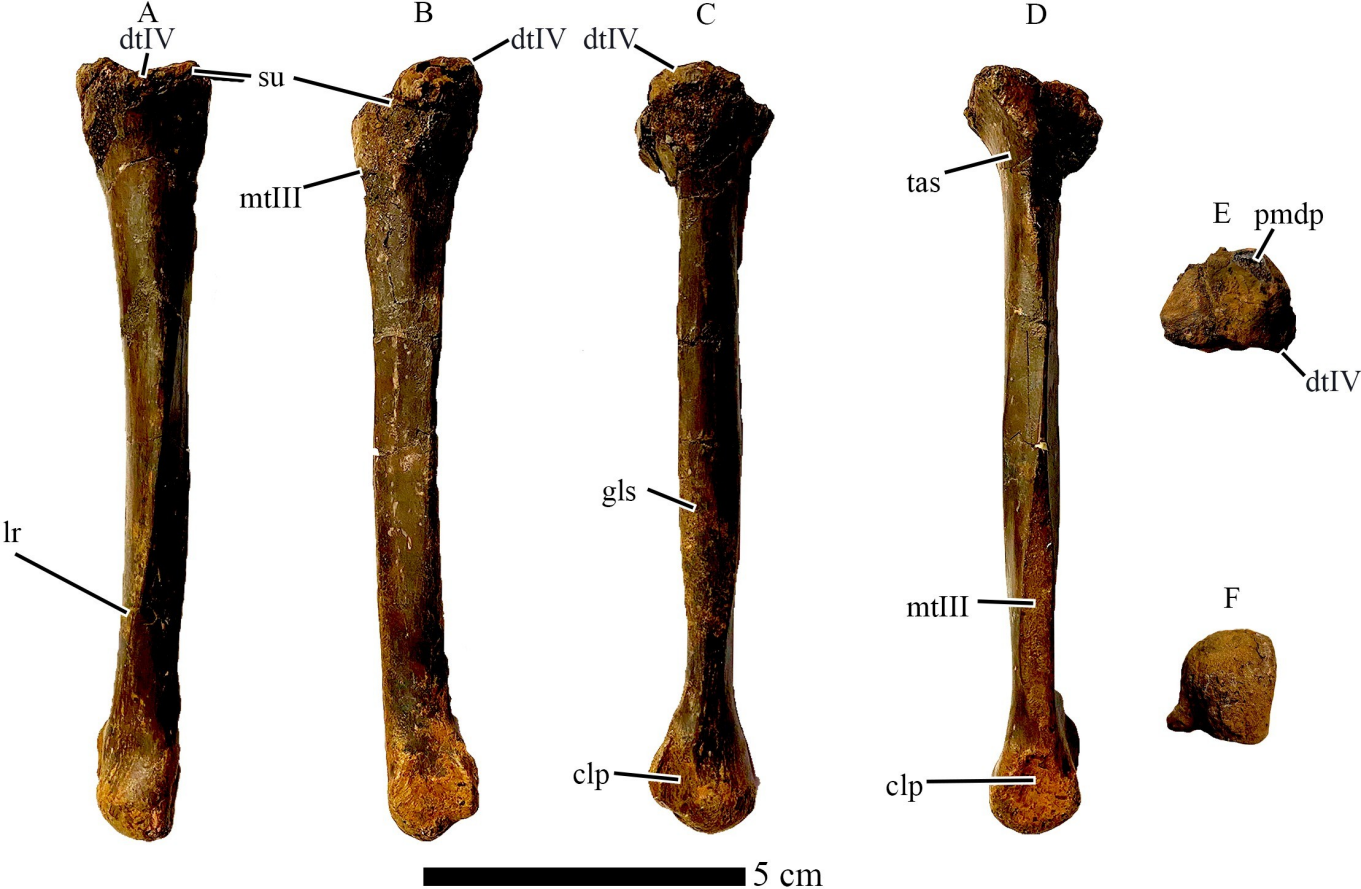
Right Metatarsal IV of *Eoneophron infernalis*. In A) Anterior, B) Posterior, C) Lateral, D) Medial, E) Proximal, and F) Distal views. Abbreviations: clp, collateral ligament pit; dtIV, distal tarsal IV; gls, M. gastrocnemius pars lateralis insertion scar; lr, longitudinal ridge; mtIII, articular surface for Metatarsal III; pmdp, broken base of proximodistal process; su, suture between distal tarsal IV and mt IV; tas, M tibialis anterior insertion scar. Scale bar = 5cm.

The two metatarsals preserved in *Eoneophron* are complete, with both proximal and distal ends intact and uncrushed shafts. The two metatarsals included with this specimen correspond to metatarsals III (Fig 4) and IV (Fig 5) from the right hindlimb. These metatarsals are shorter than those of NSM PV 21086; by 47% and 40%, respectively. Neither bones show any sign of proximal coossification, as seen in some other caenagnathids such as *Citipes elegans* and *Elmisaurus rarus* [41,43,62]. However, a small wafer of distal tarsal IV is adhered to the proximal surface of metatarsal IV, to which it has begun coossifying. The metatarsus would have been elongate, more than 300% as long as its proximal width, even when accounting for the missing metatarsal II estimated from the proportions of other caenagnathids. The metatarsus is also long relative to the proximal elements of the hindlimb, measuring 63% the length of the femur, and slightly less than half (49%) the length of the tibia. *Elmisaurus rarus* has a similarly elongate tarsometatarsus, but the proportions of the hindlimb are slightly different: in MPC-D 102/007 the tarsometatarsus is estimated at 176 mm (note there is a transcription error in Table 1 of Currie et al. 2016), roughly 72% of the femur and 51% of the tibia.

The proximal end of metatarsal III in *Eoneophron infernalis* is triangular in proximal view, with a narrow anterior wedge that would have separated metatarsals II and IV in articulation. The posterior margin of the proximal end is raised into a roughly rectangular mound, similar to, but less pronounced than the posterior protuberance in *Elmisaurus rarus* and *Citipes elegans* (but lacking in the Frenchman caenagnathid), which is formed by similar protrusions on each of the three weight-bearing metatarsals [62,64]. It is unclear whether the third distal tarsal is fused to the proximal end of metatarsal III, but there is a proximally-protruding posterior lip (Fig 4E) that may either be a portion of distal tarsal III, or posteriorly delimits the concavity into which distal tarsal III would insert. The shaft of metatarsal III is highly constricted proximally, reaching its mediolaterally narrowest point approximately a quarter of the way from the proximal articular surface. Distally along its length, it widens transversely, but it is constricted just proximal to the distal condyle (Fig 4). This is indicative of an arctometatarsalian condition, but based on its morphology, only the proximal-most portion of metatarsal III would be obscured, as is the case in *Citipes elegans*, *Chirostenotes pergracilis*, and *Elmisaurus rarus* [39,41,62]. This results in the semi-arctometatarsalian condition unique to caenagnathids among theropods. A similar condition is exhibited by the third metatarsal described by Tsujimura et al. (2021) [63], but that specimen is relatively broader transversely, whereas metatarsal III of *Eoneophron infernalis* is more gracile throughout its length. A longitudinal ridge extends distally from the wedge-like anterior edge of the proximal end, grading into the extensor surface of the metatarsal about halfway down its length. Distal to this, two longitudinal grooves on either side of the ridge merge to create a shallow concavity on the extensor surface of the metatarsal. The same is true of *cf*. *Anzu wyliei* (NSM PV 21086; [63]), but this ridge and the accompanying concavity are less pronounced. The shaft of the metatarsal flares transversely about 30% of the way from the distal end, which is similar to other caenagnathids. However, in *Eoneophron*, this flared portion is equal in width to the distal condyle, whereas it is distinctly narrower in *Citipes elegans*, *Elmisaurus rarus*, and RSM P2600.1. The distal condyle is large and slightly asymmetrical, such that the medial portion of the condyle is larger than the lateral portion, like in RSM P2600.1. The condyle is transversely wider than anteroposteriorly deep, which contrasts with *Citipes elegans* but is similar to *Chirostenotes pergracilis* and *Elmisaurus rarus* [35]. In RSM P2600.1, the distal condyle is nearly equal in transverse and anteroposterior dimensions. In metatarsal III of *Eoneophron*, as like other theropods, the medial collateral ligament pit is deeper than the lateral one. Towards the posterior (flexor) part of the condyle, the edges of the articular surface become attenuated into sharp ridges that extend and merge proximally. These are continuous with the prominent longitudinal ridges on the shaft of the metatarsal, and together they form the distinctive cross that is characteristic of most caenagnathids [39,62]. Similar ridges are present on the metatarsal III of *cf*. *Anzu wyliei*, suggesting they are not restricted to ‘elmisaurine’ caenagnathids (contra Funston et al. 2016), but in NSM PV 21086, they are more narrowly spaced, whereas in *Eoneophron infernalis*, they are separated by a wider sulcus and are closer to the medial and lateral edges of the metatarsal. Variation among caenagnathids also exists in the continuity of the cruciate ridges with the ridges of the distal condyles. In *Eoneophron infernalis*, like *Citipes elegans*, *Elmisaurus rarus* and RSM P2600.1, they are distinctly continuous, forming a pronounced X-shaped feature, whereas in TMP 1979.020.0001, the ridges are not continuous, resulting in a smooth region between these sets of ridges [41]. Overall, the third metatarsal compares well with the morphology seen in other caenagnathids and the less complete metatarsals of ROM VP 65884 [46]. Metatarsal III of *Eoneophron* shares with all other caenagnathids an anteroposteriorly flattened cross-section with a concave posterior surface, although this feature is better developed in North American caenagnathids than in *Elmisaurus rarus*. Furthermore, this posterior surface has sharp medial and lateral longitudinal ridges, continuous with lateral and medial postcondylar ridges respectively; together, these form a cross, as seen in *Anzu wyliei*, *Citipes elegans* and *Elmisaurus rarus*, but not *Chirostenotes pergracilis* [39].

Metatarsal IV is similar to those of other caenagnathids, especially *Citipes elegans* and *Elmisaurus rarus* [41,43,62]. In particular, distal tarsal IV is fused to the proximal end of the bone, as is seen in these two species [41,43,62], but the proximodorsal process of distal tarsal IV is broken (Fig 5). This contrasts with the condition in NSM PV 21055 (*cf*. *Anzu wyliei*; Tsujimura et al. 2021) [63], where the distal tarsal is not fused to the proximal end of metatarsal IV. The proximal end is the widest part of the bone, and would have contributed posteriorly to a protuberance as in *Elmisaurus rarus* [62]. In proximal view, the proximal end is roughly semicircular, with a flattened medial edge and a rounded lateral edge. The lateral edge has a slightly flattened facet that tapers distally, which marks the contact with the missing metatarsal V. In medial view, the proximal end of the metatarsal is incised by a triangular facet towards the posterior side, for articulation with metatarsal III. Anterior to this is a smaller facet that protrudes further medially. This facet would have contacted metatarsal II, obscuring the proximal end of metatarsal III in anterior view as in *Elmisaurus rarus* [62]. The facet for metatarsal II is continuous distally with a small ridge that outlines a proximal slit for *Arteria tarsalis plantaris* [62] when reunited with metatarsal III. A small rugose area is present on the anterior surface of the metatarsal just distal to the proximal expansion. Comparison with other theropods suggests that this is an insertion for the *Musculus tibialis anterior* [65,66]. The shaft of the metatarsal is anteroposteriorly compressed, not exceeding the thickness of the distal condyle in lateral and medial views. This contrasts with *Elmisaurus rarus* and *Citipes elegans*, where a posterolateral ridge is well developed. In *Eoneophron infernalis*, the posterolateral ridge does not protrude as far posteriorly, but it runs from the medial edge of the proximal articular surface, continuing distally to form the ridge ventral to the lateral ligament pit. However, this ridge does not form a pointed process on the proximal articular surface. This posterolateral ridge appears rugose and is likely the insertion scar for the *M*. *gastrocnemius pars lateralis* [66]. A second, prominent longitudinal anterior ridge twists along the shaft from the medial edge proximally towards the lateral edge distally. This second ridge is better developed than in *Citipes elegans* and *Elmisaurus rarus*, and it extends for a greater length of the shaft (three-quarters) than in the former taxa (roughly two thirds). The roughened facet for metatarsal III on the medial surface extends just over two thirds (70%) of the length of the bone, expanding distally to a point just above the distal condyle. From here, it tapers distally, and a small posteroventral extension of the facet contacts the roughened area surrounding the medial collateral ligament pit. The shaft of the metatarsal is slightly deflected laterally at the distal end, as observed in the left metatarsal IV of ROM VP 65884 [46]. The distal condyle is bulbous and nearly spherical. Both the lateral and medial collateral ligament pits are shallow, but the medial one is slightly deeper than the lateral one, which is flanked posteriorly by a wing-like ridge. The metatarsal IV of *Eoneophron infernalis* is remarkably similar to that of NSM PV 21055 (*cf*. *Anzu wyliei*; Tsujimura et al. 2021), albeit much smaller and with a relatively larger proximal end because of the posterior protuberance, and with a less bulbous distal condyle. In these features, it is more similar to the smaller-bodied *Citipes elegans* and *Elmisaurus rarus* [41,43,62].

### Histology

#### Femur

**Fig 6:**
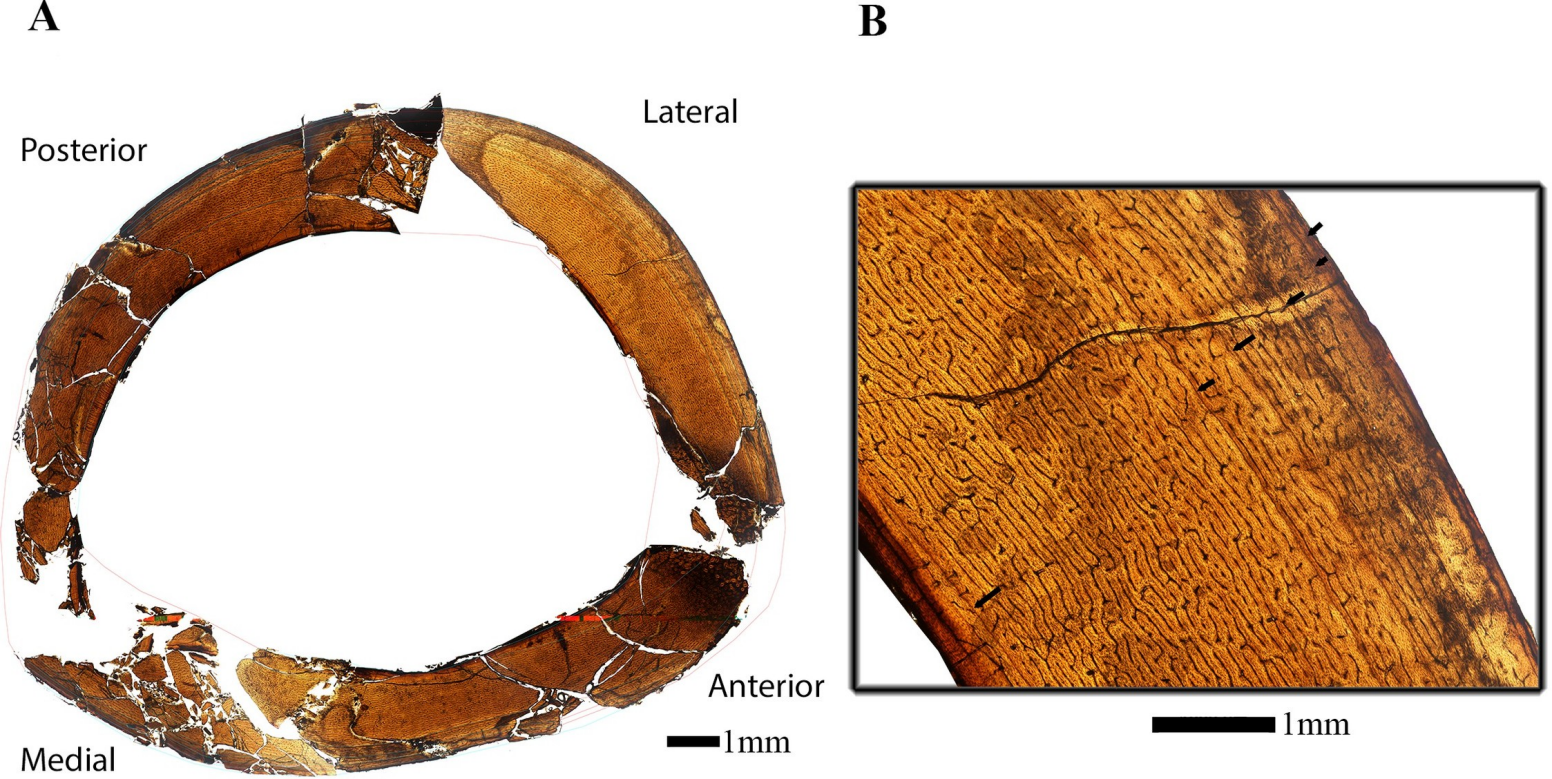
Osteological thin-section of the femur of *Eoneophron infernalis* in plain polarized light. A) Reconstructed cross section of femur with lines of arrested growth [LAGs] traced in light blue; B) Close up of cross section with black arrows denoting lines of arrested growth [LAGs]. Note the change in vascularity from the inner to outer cortex. Scale bar = 1000*μ*m.

The diaphysis was craniocaudally crushed post-burial, but cortical microstructural preservation is such that histological descriptions can still be made. There is a well-developed and complete lamellar endosteal layer, with frequent simple radial vascular canals passing between the medullary cavity and inner cortex (Fig 6). The primary cortex is fibrolamellar and vascular canals are reticular and sub-laminar. Osteocyte lacunae density is high throughout the cortex. Anterolaterally from inner to outer cortex there is a localized column of secondary osteons, likely related to a tendon enthesis. No secondary osteons are present outside of this column of remodeled bone. Six LAGs are visible. The innermost LAG is only partially present, having been resorbed by medullary expansion. Otherwise, there is no remarkable evidence of cortical drift. The zone of primary tissue between the innermost LAG and the next comprises the majority of the cortex. Thereafter, the zones between LAGs 2–6 are more closely-spaced (Table 2 and Fig 6). After the fifth LAG, vascular density is greatly decreased (Fig 6B), although osteocyte lacunae density remains high. After the fifth LAG, bone tissue is also loosely parallel-fibered. From the retrodeformed transverse thin section image, femoral shaft circumference is 105.8 mm.

**Table 2:** Comparison of relative growth for histologically sampled elements of *Eoneophron infernalis*. MC: Medullary cavity.

Element	Growth Zone	Relative % of cortex
**Femur** (undeformed)	Medullary cavity to LAG 1	Lost to MC expansion
	LAG 1 to LAG 2	6.17
	LAG 2 to LAG 3	14.20
	LAG 3 to LAG 4	3.09
	LAG 4 to LAG 5	2.47
	LAG 5 to LAG 6	1.23
	LAG 6 to outer surface	1.23
**Tibia**	Medullary cavity to LAG 1	6.40
	LAG 1 to LAG 2	11.63
	LAG 2 to LAG 3	2.33
	LAG 3 to LAG 4	2.91
	LAG 4 to LAG 5	1.74
	LAG 5 to LAG 6 (not traceable)	0.58
	LAG 6 to outer surface	0.58
**Metatarsal IV**	Medullary cavity to LAG 1	4.91
	LAG 1 to LAG 2	31.89
	LAG 2 to LAG 3	13.54
*	LAG 3 to LAG 4	2.06
*	LAG 4 to LAG 5	2.55
*	LAG 5 to LAG 6	2.16
	LAG 6 to outer surface	1.57

*Some estimation required to trace LAG.

#### Tibia

**Fig 7:**
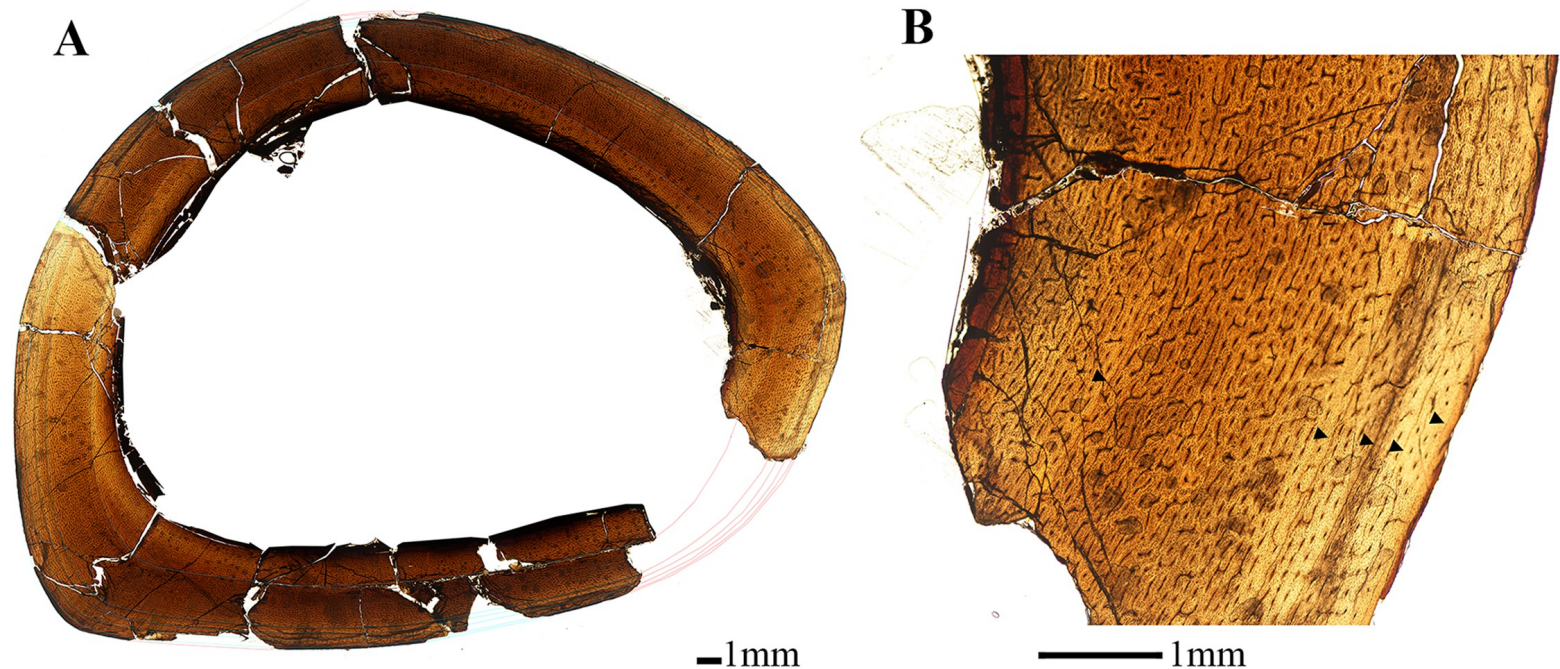
Osteological thin section of right tibia of *Eoneophron infernalis* in plain polarized light. A) Reconstructed cross section of tibia with lines of arrested growth [LAGs] traced in light blue; B) Close up of cross section with black arrows denoting lines of arrested growth [LAGs]. Note the change in vascularity from the inner to outer cortex. Scale bar = 1000*μ*m.

As with the femur, the tibia diaphysis was anteroposteriorly crushed post-burial, but a histological description is still possible. The lamellar endosteal layer is well-developed, with frequent simple radial vascular canals passing between the medullary cavity and inner cortex (Fig 7). The primary cortex is fibrolamellar and vascular canals within the inner and mid-cortex are a combination of reticular and longitudinal (Fig 7B), with frequent short radial anastomoses. Longitudinal vascularity dominates within the outer cortex. No secondary osteons are observed. The first visible LAG is located within the mid-cortex, and the tissue from the endosteal surface to the second LAG comprises the majority of the cortex (Table 2). The first LAG is only partially visible, having been largely eroded by medullary cavity expansion. Cortical drift, however, is negligible. The zones between LAGs 2–6 towards the periosteal surface are markedly thinner (Fig 7). Vascular density is also reduced in these last four zones, but only slightly. The sixth LAG is so close to the periosteal surface that it often merges with it. From the retrodeformed thin section image, transverse section tibia circumference is 106.8 mm.

#### Metatarsal IV

**Fig 8:**
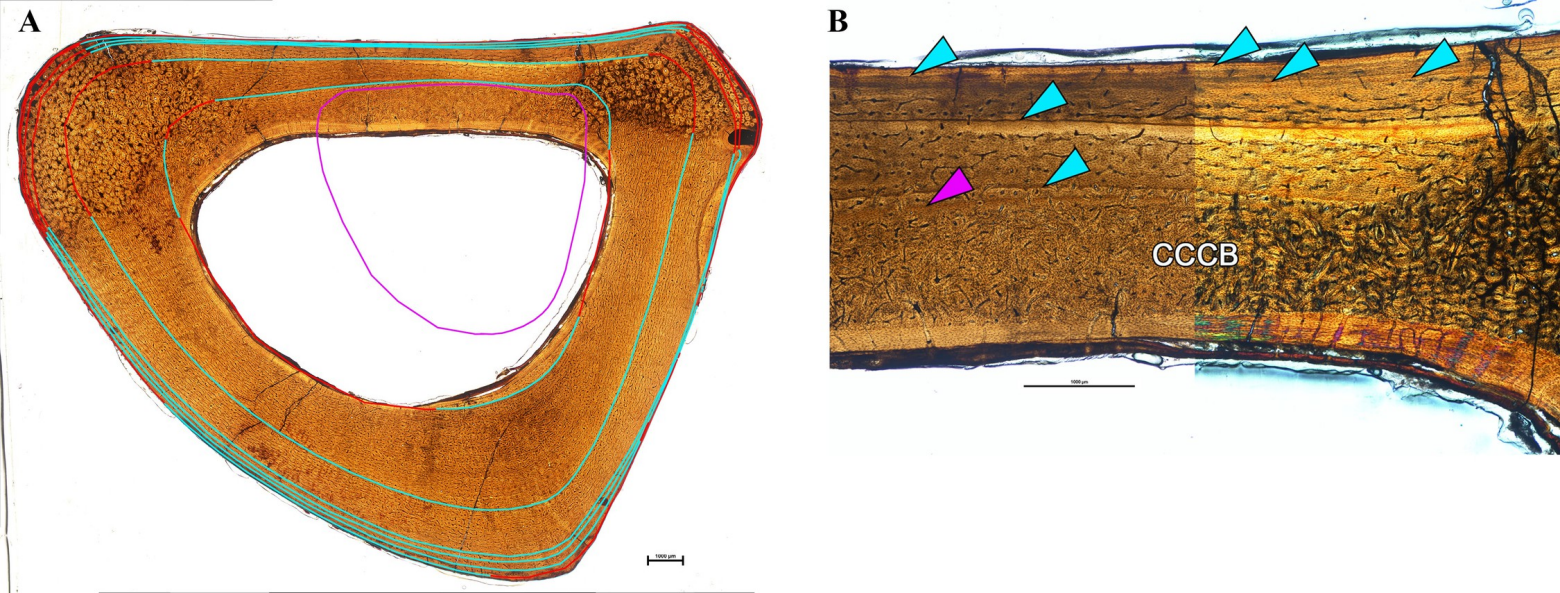
Osteological thin section of right Metatarsal IV of *Eoneophron infernalis* in plain polarized light. A) Cross section of Metatarsal IV with lines of arrested growth [LAGs] traced in light blue, with area missing due to modeling or medullary cavity expansion in red, and putative neonatal line in purple B) Close up of posterior side where CCCB, a possible neonatal line (purple arrows), and six LAGs are observed (blue arrows). Imaged in plane polarized light on the left, circularly polarized light on the right. Note the change in vascularity from the inner to outer cortex. Scale bar = 1000*μ*m.

There is a well-developed lamellar endosteal layer, and occasional simple radial vascular canals pass between the medullary cavity and primary cortex (Fig 8). Primary tissue from inner to outer cortex is fibrolamellar with a high density of osteocyte lacunae. Vascular canal density is somewhat higher in the innermost cortex compared with the mid- and outer cortex. Anteromedially, canals are primarily a combination of longitudinal and reticular, and are largely sub-laminar elsewhere. Posteriorly within the innermost cortex there is a crescent of compact coarse cancellous bone (CCCB) [67] between the lamellar endosteal layer and the periosteally-derived mid-cortex. The CCCB grades into a thin annulus of well-organized avascular tissue, consisting of numerous flattened osteocyte lacunae. This annulus is truncated posteromedially and posterolaterally by medullary cavity resorption. Beyond the annulus, cortical tissue is fibrolamellar, and vascular density is high. CCCB results when spaces between spongy trabecular bone are infilled by additional bone to result in compact tissue [67]. This process occurs at the metaphyseal regions of long bones during endochondral ossification [67]. Embryonic CCCB is sometimes found within the shaft of a long bone resulting from subsequent diaphyseal lengthening [68]. A change in vascular density beyond the annulus [69–72] and the close proximity of a periosteally-derived annulus to the CCCB are morphologically consistent with a neonatal line (Fig 8B). The neonatal line, found in the bones and teeth of amniote tetrapods, results from a brief pause in growth occurring around the time of hatching or birth [72]. The neonatal line provides an estimate of perinatal bone circumference [72], which for metatarsal IV is 26.1 mm.

On the anteromedial and anterolateral sides, a localized column of secondary osteons spans from inner to outer cortex, likely related to ligamentous entheses. Beyond these columns, no secondary osteons are present within the cortex. A nutrient foramen is present near the periosteal surface on the medial side.

Within the outer cortex, vascular density is decreased, consisting of scattered longitudinal and reticular vascular canals (Fig 8B). Osteocyte lacunae density remains high in the outermost cortex on the anterior side, and is less dense with more flattened lacunae on the posterior side. Beyond the annulus bounding the inner-cortical CCCB, six LAGs are present. Three widely-spaced LAGs are visible within the cortex, with the innermost only partially visible due to erosion from medullary cavity expansion. LAG spacing is then abruptly decreased between the final three LAGs nearest the periosteal surface. LAGs 4–6 are traceable, but merge with the periosteal surface in places (Table 2 and Fig 8). Although the primary tissue within these outer three zones is fibrolamellar on the anterior side, the tissue is parallel-fibered to lamellar on the posterior side. An overlay of LAG tracings reveals very little cortical drift over ontogeny after the first year of life. Transverse thin section circumference is 64.2 mm.

### Mass estimation

The reconstructed femoral circumference of 105.8mm for *Eoneophron infernalis* predicts a body mass of 78 kg based on the equation of Campione et al. [56], with a range from 58.9–98.1 kg. This is far less than predicted for the much larger CM 78000, which, using the same methods, weighed between 202 and 342 kg. However, the body mass estimate for CM 96523 is only slightly larger than those of other mid-sized caenagnathids like *Anomalipes* (67 kg), *Apatoraptor* (63 kg), *Chirostenotes* (67 kg), and *Elmisaurus* (65 kg) [59].

### Phylogeny

**Fig 9:**
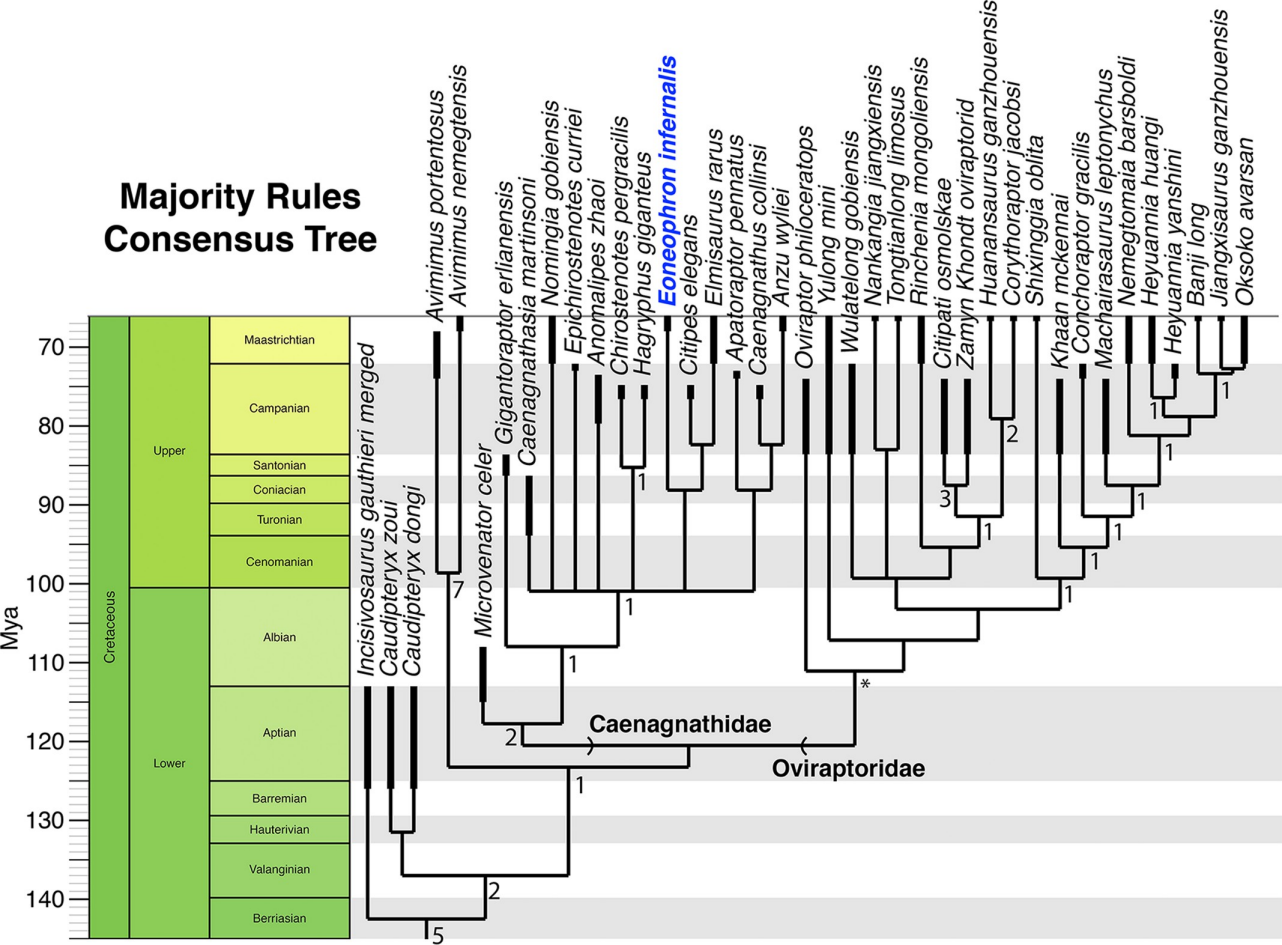
Time-calibrated majority-rules consensus tree showing the position of *Eoneophron infernalis*. Tree is trimmed to Oviraptorosauria. Numbers next to nodes indicate Bremer support values in the strict consensus tree; nodes without numbers were not recovered in the strict consensus. * indicates a node that never collapsed under the Bremer analysis (i.e., it is exceedingly well-supported).

Our phylogenetic analysis returned 504 most parsimonious trees with a tree length of 648 steps (Fig 9). The topology of the strict consensus is similar to previous analyses of oviraptorosaur phylogeny, in dividing oviraptorids into two main subfamilies (although with a large basal polytomy), and recovering *Eoneophron* among derived caenagnathids within a large polytomy. Bremer support for the major nodes in Oviraptorosauria is strong, but internal support is weaker, as expected for a dataset with a high proportion of missing data. In the majority-rules consensus, *Eoneophron infernalis* is recovered as a sister to the clade of *Elmisaurus* and *Citipes*, which is reasonable because of the interpretation of the fusion of distal tarsal IV to metatarsal IV. In any case, phylogenetic analysis strongly supports the identification of *Eoneophron* as a caenagnathid, although with expectedly low resolution considering the fragmentary preservation of the holotype specimen and most other known caenagnathid material.

## Discussion

### Morphology

The morphology of *Eoneophron infernalis* is broadly consistent with that of other caenagnathids, but it provides additional information on their proportions and variation. There is a negative allometric relationship between tibia and femur length in Oviraptorosauria [73]; *E*. *infernalis* proportions are scaled similarly to those of *Anzu wyliei*, *Chirostenotes pergracilis*, *Elmisaurus rarus*, and *Gigantoraptor* [5,8,12,62], suggesting that this negative allometry is conserved across caenagnathids.

Several discrete morphological features indicate that *Eoneophron infernalis* is distinct from *Anzu wyliei* and other caenagnathids. Histological data also supports this separation (see below), which indicate a smaller adult body size than *Anzu wyliei*. The head of the femur of *E*. *infernalis* meets the shaft at an oblique angle, rather than extending perpendicular, as is the case in *Anzu wyliei* [12] and most other caenagnathids. Furthermore, the femoral condyles appear to be relatively smaller in *E*. *infernalis*, but it is unclear whether this is attributable to allometry. The accessory trochanteric crest of *Eoneophron infernalis* is much smaller than that of *Anzu wyliei*, *Elmisaurus rarus*, and *Nomingia gobiensis*. The tibia has a more proximally restricted cnemial crest than in *Anzu wyliei*, but it is otherwise similar in morphology to most oviraptorosaurs [73], although it has a less well-developed post-fibular flange than most caenagnathids. *Eoneophron infernalis* differs from *Anzu wyliei* in the extension of the astragalus to reach the lateral margin of the tarsus, a feature that has sometimes been used to distinguish caenagnathids from oviraptorids [61], but is likewise present in RSM P2600.1. Although the referral of metatarsals to *Anzu wyliei* remains equivocal, *E*. *infernalis* differs in several respects from the larger metatarsals from the Hell Creek Formation. In particular, the presence of posterior protuberances on metatarsals III and IV is reminiscent of the tripartite protuberance that characterizes *Citipes elegans* and *Elmisaurus rarus*, and it is likely that *Eoneophron infernalis* shared this feature. These protuberances are absent on the proximal ends of the metatarsals referred to *cf*. *Anzu wyliei*. Similarly, metatarsals referred to *cf*. *Anzu wyliei* lack evidence of fusion between distal tarsal IV and metatarsal IV, or a pointed process on the proximolateral edge of metatarsal IV.

While the gross morphology of *Eoneophron infernalis* elements is generally similar to other caenagnathids, its hindlimb is remarkable for the extensive fusion of the bones. Fusion of the astragalus with the calcaneum and fusion of the distal tarsals with the proximal metatarsals have been described previously in caenagnathids, but fusion between the astragalocalcaneum and the tibia has not. Within Oviraptorosauria, only avimimids are known to exhibit fused tibiotarsi [24], and they also exhibit extensive fusion elsewhere in the skeleton, including the braincase, manus, and pelvis. It is noteworthy that in several specimens of *Elmisaurus rarus*, the tarsometatarsi are coossified, but the associated tibiotarsi are not: in each the tibiae show no signs of fusion to the astragalocalcanea, which are missing. Thus, although there is clearly an ontogenetic aspect to fusion of the tibiotarsus, it appears that the fusion of the tibiotarsus in *Eoneophron infernalis* is unique to this taxon within Caenagnathoidea, because other specimens at presumably equivalent ontogenetic stages do not show this feature [3,32].

Together, these morphological features distinguish CM 96523 from *Anzu wyliei* and other caenagnathids known from the Late Cretaceous of North America. Considering that histological data show that this specimen was approaching adult body size and may have already reached sexual maturity (see below), the data support the establishment of a new taxon for this material, *Eoneophron infernalis*. This taxon can be distinguished by a unique combination of features of the hindlimb, as well as two autapomorphies: fusion of the astragalocalcaneum and tibiotarsus at maturity and a long, sharp longitudinal ridge on the anterior surface of metatarsal IV.

### Histology

CM 96523 contributes to a growing body of caenagnathid long bone histoanalyses [39,41,46,51,73,74], but is the first published assessment of caenagnathid femoral histology. The histology of the femur, tibia, and metatarsal IV are congruent, suggesting that the ontogenetic signal they provide is reliable. The femur, tibia, and metatarsal IV of *Eoneophron infernalis* each record six LAGs. Although the large medullary cavities of each bone leave the possibility that one or more LAGs from earlier ontogeny were obliterated by medullary cavity expansion, the presence of a putative neonatal line followed by six LAGs in metatarsal IV suggests the death of this individual occurred during its sixth year of life. Because the sixth LAG is so close to the periosteal surface and often merges with the surface in each bone examined, death probably occurred shortly after this individual emerged from its annual growth hiatus.

LAG spacing reveals that the majority of bone apposition and body size increase happened during the first two years of age, based on femur and tibia histology. After the second LAG, there is a marked shift to closely spaced LAGs, comprising the remainder of the outer cortex. This inflection in apposition rate may correspond to sexual maturity, as hypothesized for other dinosaur groups including oviraptorosaurs [75–77]. Interestingly, apposition rate inflection occurred after the third year within metatarsal IV, rather than in the second year as observed in the femur and tibia. The cortex of the femur and tibia is thinner than that of metatarsal IV, which does indicate differing rates of medullary cavity enlargement. Indeed, that the putative neonatal line is recorded in metatarsal IV and not in the femur or tibia is strong evidence that medullary expansion rate was lower in the former. However, because the cortex of metatarsal IV is thicker and still records only six LAGs, differences in apposition rate inflection reflect individual bone growth trajectories rather than mismatched LAG counts due to medullary resorption.

The tibia histology of *Eoneophron infernalis* differs from that of a previously described indeterminate caenagnathid (UALVP 57349) from the Campanian-early Maastrichtian Horseshoe Canyon Formation in Alberta, Canada [73]. The histology of UALVP 57349 suggests this small individual (tibia length of 210 mm) was a young juvenile. Its bone tissue is entirely reticular and plexiform fibrolamellar, with open vascular canals at the periosteal surface, no secondary osteons, and an avascular endosteal lamellar layer. A band of parallel-fibered tissue within the cortex may be an annulus, suggesting it was at least one year of age [73]. The larger tibia of *E*. *infernalis* (480mm) described here is also entirely fibrolamellar, but vascular orientation is reticular to sub-laminar, has a vascularized lamellar endosteal layer, and there are six well-defined LAGs within the cortex. Beginning with the fourth LAG, zonal spacing between LAGs is markedly decreased towards the periosteal surface, and the vascular canals are not open at the bone surface. The histology of UALVP 57349 and *E*. *infernalis* show that the tibia of each was still growing when the individuals died. However, the histology of *E*. *infernalis* suggests it was relatively older (six LAGs compared to a single annulus) and growth was approaching an asymptote, whereas UALVP 57349 shows no indication of decreasing annual apposition rate.

The histology of *Eoneophron infernalis* also differs from the fragmentary tibia of ROM VP 65884, referred to *Anzu wyliei* [46], from the Maastrichtian Hell Creek Formation of Montana. Although the primary tissue in both tibiae is fibrolamellar, the vascularity in ROM VP 65884 ranges from reticular within the inner cortex to plexiform in the outer cortex, while that of *E*. *infernalis* is a combination of reticular and longitudinal for most of the cortex to predominantly longitudinal in the outermost cortex. ROM VP 65884 preserves seven LAGs, with a noticeable decrease in zonal spacing occurring after the third LAG. The tibia of *E*. *infernalis* preserves only six LAGs, with a pronounced decrease in zonal thickness occurring after the second LAG. Finally, although zonal spacing is decreased within the outer zones of the larger tibia of ROM VP 65884, tissue remains well-vascularized to the periosteal surface and there is no indication an EFS is present. This differs from *E*. *infernalis*, which exhibits reduced, longitudinal vascularity within the outermost cortical zones.

Vascular orientation can be compared to determine relative growth rates [78]. Longitudinal vascular canals within the outermost zones of the tibia in *Eoneophron infernalis* indicate a slower annual growth rate than the plexiform vascularity observed in tibia ROM VP 65884, referred to *A*. *wyliei*. Although individual variation in growth cannot be discounted (e.g., Woodward et al. 2015 [79]), it appears that *E*. *infernalis* would have attained a smaller asymptotic body size than reported for specimens of *A*. *wyliei* [12,46].

The metatarsal IV histology of CM 96523 resembles that of the Campanian-aged Dinosaur Park Formation *Citipes elegans* metatarsal IV (UALVP 59606) described previously by Funston et al. (2020) [74]. In both, the bone tissue is reticular to longitudinal fibrolamellar, the lamellar endosteal layer is pierced by vascular canals, and the cortex preserves six LAGs. The two metatarsals differ in that the LAG spacing in *C*. *elegans* metatarsal IV becomes reduced after the second LAG, while this occurs after the third LAG in CM 96523. Also, the metatarsal IV of *C*. *elegans* (total length of 146 mm) contained secondary osteons, and the closely spaced LAGs within avascular outermost cortex was interpreted as an EFS and signaling skeletal maturity [74]. In contrast, the zones between the closely spaced outermost LAGs in metatarsal IV of *E*. *infernalis* (total length of 233mm) remain vascularized with high osteocyte lacuna density, suggesting that this larger metatarsal was approaching asymptotic size but was still growing in length at death.

### Paleoecological and diagnostic implications

The number of caenagnathid taxa in the Hell Creek Formation is uncertain. Varricchio (2001) described a partial pes of a small caenagnathid (MOR 752) he referred to “*Elmisaurus*’’ *elegans* (now *Citipes elegans*) on the basis of an anterolateral process proximal to the distal condyle of metatarsal IV, for *Musculus tibialis cranialis* [39,43]. At the time, the presence of a larger caenagnathid in the Hell Creek Formation was known [38,80], but it was not formally described until 2014, as *Anzu wyliei* [12]. There is, however, the lack of overlap of the elements described by Varricchio (2001) and Lamanna et al. (2014), and thus it is unclear whether they could represent ontogimorphs of the same taxon. *Eoneophron infernalis* complicates this issue somewhat, as it is nearly double the size of MOR 752 but shows clear signs of skeletal maturity. It is thus unclear whether these specimens indicate the presence of two taxa—in which case MOR 752 is a juvenile of either *Anzu wyliei* or *Eoneophron*—or three taxa, in which case each represents a distinct taxon. However, some evidence suggests that the latter situation may be more likely. For example, the metatarsal II referred to *cf*. *Anzu wyliei* by Cullen et al. (2020) lacks the anterolateral process proximal to the distal condyle described for MOR 752, which also distinguishes it from *Citipes elegans* and *Elmisaurus rarus*. As described by Currie et al. (2016) and Funston et al. (2016), the prominent attachment sites for *M*. *tibialis cranialis* in *Citipes elegans* and *Elmisaurus rarus* consistently manifest as both the anterolateral process on metatarsal II and an anteromedial process on metatarsal IV. This suggests that, were it preserved, metatarsal IV of the taxon represented by MOR 752 could be expected to have a prominent anteromedial process proximal to the distal condyle. However, this is not the case in *Eoneophron infernalis*, despite nearing skeletal maturity, tentatively suggesting that it is distinct from MOR 752. As muscular insertions might be expected to become better developed allometrically and ontogenetically, this casts doubt on the idea that MOR 752 is a juvenile individual of either *E*. *infernalis* or *Anzu wyliei*. Nonetheless, as MOR 752 is fragmentary, its ontogenetic status is unknown, and the ontogeny of muscle attachments in caenagnathids is poorly understood, caution is warranted in ascribing the remains to different taxa.

**Fig 10:**
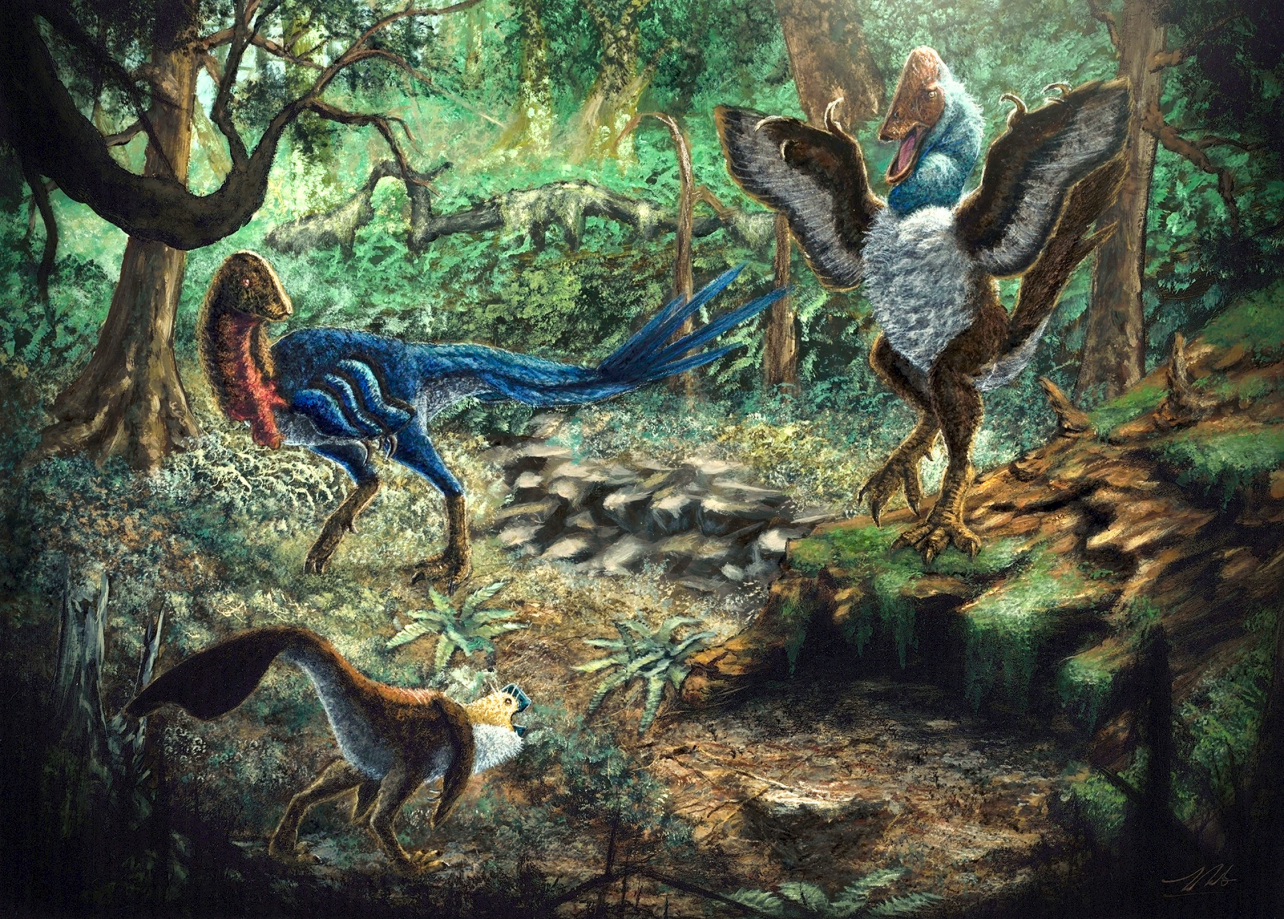
Artist’s depiction of *Eoneophron infernalis* (top left), MOR 752 (bottom left), and *Anzu wyliei* (right) in the Hell Creek Formation. Illustration by Zubin Erik Dutta.

The presence of either two or three caenagnathid taxa in the Hell Creek Formation indicates nevertheless that multiple species of caenagnathid coexisted during the end-Maastrichtian (Fig 10), and highlights palaeohistology as a key source of information on the diversity of caenagnathids across North America [35,41,46,51,52,74]. Coexistence of multiple caenagnathids in the Hell Creek Formation is not unexpected, given that the Campanian Dinosaur Park Formation also preserves three caenagnathid taxa within the same ecosystem, each of a different adult size [41], and the Horseshoe Canyon Formation likewise preserves two differently-sized taxa [36,47]. Although tentative, two sizes of caenagnathid also appear to be present in the Maastrichtian Frenchman Formation of Saskatchewan, Canada [39,81]. In the Dinosaur Park and Frenchman formations, the largest species approached *Anzu wyliei* in size [40,41,81]. However, there were also smaller taxa like *Citipes elegans*, and in the Dinosaur Park Formation, an intermediately-sized species is present: *Chirostenotes pergracilis*. *Eoneophron infernalis* appears closest to *Chirostenotes* in size, whereas MOR 752 is similar in size to *Citipes elegans* [43]. Tibiae and femora have not yet been unambiguously referred to *Citipes*, so its proportions are difficult to estimate, but it was clearly the smallest caenagnathid within the Dinosaur Park Formation [41]. Despite being similar in body mass to *Chirostenotes pergracilis*, in many features *E*. *infernalis* is more similar to the smaller *Citipes elegans* and *Elmisaurus rarus*, an affinity borne out by the phylogenetic analysis conducted here. Like in the Dinosaur Park Formation [41], the differing adult body masses of caenagnathid taxa in the Hell Creek Formation suggests that size was an important factor in the coexistence of multiple caenagnathids in the same ecosystems. However, it is interesting to note that the intermediately-sized taxa, *Chirostenotes pergracilis* and *Eoneophron infernalis*, differed considerably in metatarsal morphology. Thus, it tentatively appears that body size niches were not necessarily restricted to taxa with a particular set of adaptations. Unfortunately, little is known about the ecology of caenagnathids, so it is difficult to speculate on the impacts of the fused limb elements of *E*. *infernalis* on its behavior. Whereas it is possible that the fusion of proximal tarsals to metatarsus better allowed *E*. *infernalis* to handle stresses associated with cursorial locomotion, evidence from the pelvic regions suggests that caenagnathids of similar proportions may have had divergent locomotory behaviors [82]. Similarly, the grasping adaptations of the caenagnathid pes [80] may have served a role in locomotion, prey capture, or both, and thus the morphology of the metatarsus may reflect a mosaic of selective pressures.

The unexpected histological results further highlight the importance of taking histological data into account when assigning taxonomic identities to incomplete theropod remains, reinforcing the point originally made by Cullen et al. 2020 [46]. Likewise, Cullen et al. 2020 raise concerns regarding the sampling and taxonomic stability of even well-sampled geological formations. The presence of cryptic diversity, like that revealed here, may have important ramifications for estimations of dinosaur diversity, environmental sensitivity, community structure, and ecosystem vulnerability in Late Cretaceous ecosystems, especially those leading up to the end-Cretaceous extinction.

## Conclusions

CM 96523 represents one of the few instances of associated caenagnathid hindlimbs, and exhibits unique proportions, combinations of features, and coossifications between elements. In tandem with histological data indicating that this animal was of subadult or adult status at the time of its death, this suggests that it represent a new taxon, *Eoneophron infernalis*. This smaller caenagnathid from the Hell Creek Formation has implications for the ecology and diversity of caenagnathids in the end-Maastrichtian. As many as three taxa of varying body size may have inhabited the Maastrichtian ecosystems of the Hell Creek Formation, but like in other regions, a poor fossil record makes untangling the taxonomy of these species problematic. The ecology of caenagnathids likewise remains poorly understood, but *E*. *infernalis* expands the range of morphological variation within the family and suggests that caenagnathid diversity in Laurasia remained largely stable through the Campanian-Maastrichtian, and these dinosaurs remained successful components of Laurasian ecosystems until the K-Pg extinction.

## Supporting information

S1 File NEXUS phylogenetic data used in the study. (NEX)

S2 File TNT phylogenetic data used in the study. (TNT)

S3 File PHYLIP phylogenetic data used in the study. (PHY)

S4 File Phylogenetic tree generated from data used in the study. (TRE)

## References

[1] Osmólska H. Oviraptorosauria. In: Weishampel D, Dodson P, Osmólska H, editors. The dinosauria. Berkeley, CA: University of California Press; 2004. pp. 165–183.

[2] Funston GF, Chinzorig T, Tsogtbaatar K, Kobayashi Y, Sullivan C, Currie PJ. A new two-fingered dinosaur sheds light on the radiation of Oviraptorosauria. R Soc Open Sci. 2020;7: 201184. doi: 10.1098/rsos.201184 33204472 PMC7657903

[3] Qiang J, Currie PJ, Norell MA, Shu-An J. Two feathered dinosaurs from northeastern China. Nature. 1998;393: 753–761. doi: 10.1038/31635

[4] Lü J, Currie PJ, Xu L, Zhang X, Pu H, Jia S. Chicken-sized oviraptorid dinosaurs from central China and their ontogenetic implications. Naturwissenschaften. 2013;100: 165–175. doi: 10.1007/s00114-012-1007-0 23314810

[5] Xu X, Tan Q, Wang J, Zhao X, Tan L. A gigantic bird-like dinosaur from the Late Cretaceous of China. Nature. 2007 [cited 28 Feb 2017]. doi: 10.1038/nature05849 17565365

[6] Zhou Z-H, Wang X-L, Zhang F-C, Xu X. Important features of *Caudipteryx* ‐ evidence from two nearly complete new specimens. Vertebr Palasiat. 2000;38: 242–254.

[7] Xu X, Zheng X, You H. Exceptional dinosaur fossils show ontogenetic development of early feathers. Nature. 2010;464: 1338–1341. doi: 10.1038/nature08965 20428169

[8] Funston GF, Currie PJ. New material of *Chirostenotes pergracilis* (Theropoda, Oviraptorosauria) from the Campanian Dinosaur Park Formation of Alberta, Canada. Hist Biol. 2020; 1–15. doi: 10.1080/08912963.2020.1726908

[9] Kurzanov S. Structural characteristics of the fore limbs of *Avimimus*. Paleontol J. 1982;1982: 108–112.

[10] Barsbold R, Currie PJ, Myhrvold NP, Osmólska H, Tsogtbaatar K, Watabe M. A pygostyle from a non-avian theropod. Nature. 2000;403: 155–156. doi: 10.1038/35003103 10646588

[11] Barsbold R, Osmólska H, Watabe M, Currie PJ, Tsogtbaatar K. A new oviraptorosaur (Dinosauria, Theropoda) from Mongolia: the first dinosaur with a pygostyle. Acta Palaeontol Pol. 2000;45: 97–106.

[12] Lamanna MC, Sues H-D, Schachner ER, Lyson TR. A new large-bodied oviraptorosaurian theropod dinosaur from the latest Cretaceous of western North America. PLoS One. 2014;9: e92022. doi: 10.1371/journal.pone.0092022 24647078 PMC3960162

[13] Pittman M, O’Connor J, Field D, Turner A, Ma W, Makovicky P, et al. Chapter 1: Pennaraptoran Systematics. 2020. pp. 7–36.

[14] Pittman M, O’Connor J, Tse E, Makovicky P, Field D, Ma W, et al. The Fossil Record of Mesozoic and Paleocene Pennaraptorans. Bull Am Mus Nat Hist. 2020;440: 37–96.

[15] Norell MA, Clark JM, Chiappe LM, Dashzeveg D. A nesting dinosaur. Nature. 1995;378: 774–776.

[16] Yang Tzu-Ruei Sander P. Martin. The reproductive biology of oviraptorosaurs: a synthesis. Geol Soc Lond Spec Publ. 2022;521: 19–34. doi: 10.1144/SP521-2021-181

[17] Maryańska T, Osmólska H, Wolsan M. Avialan status for Oviraptorosauria. Acta Palaeontol Pol. 2002;47: 97–116.

[18] Bi S, Amiot R, Peyre de Fabrègues C, Pittman M, Lamanna MC, Yu Y, et al. An oviraptorid preserved atop an embryo-bearing egg clutch sheds light on the reproductive biology of non-avialan theropod dinosaurs. Sci Bull. 2021;66: 947–954. doi: 10.1016/j.scib.2020.12.018 36654242

[19] Norell MA, Balanoff AM, Barta DE, Erickson GM. A second specimen of *Citipati osmolskae* associated with a nest of eggs from Ukhaa Tolgod, Omnogov Aimag, Mongolia. Am Mus Novit. 2018;3899: 44.

[20] Balanoff AM, Bever GS, Rowe TB, Norell MA. Evolutionary origins of the avian brain. Nature. 2013;501: 93–96. doi: 10.1038/nature12424 23903660

[21] Xu X, Cheng Y-N, Wang X-L, Chang C-H. An unusual oviraptorosaurian dinosaur from China. Nature. 2002;419: 291–293. doi: 10.1038/nature00966 12239565

[22] Smith D. Neues Jahrb Für Geol Palӓontologie Abh. 1992;186: 365–388.

[23] Zhou Z-H, Wang X-L. A new species of *Caudipteryx* from the Yixian Formation of Liaoning, Northeast China. Vertebr Palasiat. 2000;38.

[24] Funston GF, Currie PJ, Ryan MJ, Dong Z-M. Birdlike growth and mixed-age flocks in avimimids (Theropoda, Oviraptorosauria). Sci Rep. 2019;9: 18816. doi: 10.1038/s41598-019-55038-5 31827127 PMC6906459

[25] Funston G, Currie P, Eberth D, Ryan M, Chinzorig T, Badamgarav D, et al. The first oviraptorosaur (Dinosauria: Theropoda) bonebed: Evidence of gregarious behaviour in a maniraptoran theropod. Sci Rep. 2016;6: 35782. doi: 10.1038/srep35782 27767062 PMC5073311

[26] Osborn H. Three new Theropoda, Protoceratops zone, central Mongolia. Am Mus Novit. 1924;144: 1–12.

[27] Dong Z-M, Currie PJ. On the discovery of an oviraptorid skeleton on a nest of eggs at Bayan Mandahu, Inner Mongolia, People’s Republic of China. Can J Earth Sci. 1996;33: 631–636. doi: 10.1139/e96-046

[28] Clark JM, Norell M, Chiappe LM, Project M-AMP, Akademi MSU. An oviraptorid skeleton from the late Cretaceous of Ukhaa Tolgod, Mongolia, preserved in an avianlike brooding position over an oviraptorid nest. American Museum novitates; no. 3265. 1999 [cited 18 Nov 2020]. Available: http://digitallibrary.amnh.org/handle/2246/3102.

[29] Varricchio DJ, Moore JR, Erickson GM, Norell MA, Jackson FD, Borkowski JJ. Avian paternal care had dinosaur origin. Science. 2008;322: 1826–1828. doi: 10.1126/science.1163245 19095938

[30] Fanti F, Currie PJ, Badamgarav D. New Specimens of *Nemegtomaia* from the Baruungoyot and Nemegt Formations (Late Cretaceous) of Mongolia. PLOS ONE. 2012;7: e31330. doi: 10.1371/journal.pone.0031330 22347465 PMC3275628

[31] Simon DJ, Varricchio DJ, Jackson FD, Robison S. Giant theropod eggs from the Aptian-Cenomanian Wayan Formation of Idaho: Taxonomic, paleogeographic and reproductive implications. J Vertebr Paleontol Programs Abstr. 2012;32: 172.

[32] Sato T, Cheng Y-N, Wu X, Zelenitsky DK, Hsiao Y. A pair of shelled eggs inside a female dinosaur. Science. 2005;308: 375–375. doi: 10.1126/science.1110578 15831749

[33] Osmólska H. Evidence on relation of brain to endocranial cavity in oviraptorid dinosaurs. Acta Palaeontol Pol. 2004;49: 321–324.

[34] Kundrát M. Avian-like attributes of a virtual brain model of the oviraptorid theropod *Conchoraptor gracilis*. Naturwissenschaften. 2007;94: 499–504. doi: 10.1007/s00114-007-0219-1 17277940

[35] Currie PJ, Russell DA. Osteology and relationships of *Chirostenotes pergracilis* (Saurischia, Theropoda) from the Judith River (Oldman) Formation of Alberta, Canada. Can J Earth Sci. 1988;25: 972–986. doi: 10.1139/e88-097

[36] Sues H-D. On *Chirostenotes*, a Late Cretaceous Oviraptorosaur (Dinosauria: Theropoda) from Western North America. J Vertebr Paleontol. 1997;17: 698–716.

[37] Zanno LE, Sampson SD. A new oviraptorosaur (Theropoda, Maniraptora) from the Late Cretaceous (Campanian) of Utah. J Vertebr Paleontol. 2005;25: 897–904.

[38] Currie PJ, Godfrey SJ, Nessov L. New caenagnathid (Dinosauria: Theropoda) specimens from the Upper Cretaceous of North America and Asia. Can J Earth Sci. 1993;30: 2255–2272. doi: 10.1139/e93-196

[39] Funston GF, Currie PJ, Burns ME. New elmisaurine specimens from North America and their relationship to the Mongolian *Elmisaurus rarus*. Acta Palaeontol Pol. 2016;61: 159–173. doi: 10.4202/app.00129.2014

[40] Funston GF, Currie PJ. A previously undescribed caenagnathid mandible from the late Campanian of Alberta, and insights into the diet of *Chirostenotes pergracilis* (Dinosauria: Oviraptorosauria). Sues H, editor. Can J Earth Sci. 2014;51: 156–165. doi: 10.1139/cjes-2013-0186

[41] Funston G. Caenagnathids of the Dinosaur Park Formation (Campanian) of Alberta, Canada: anatomy, osteohistology, taxonomy, and evolution. Vertebr Anat Morphol Palaeontol. 2020;8: 105–153. doi: 10.18435/vamp29362

[42] Funston GF, Persons IV S, Bradley GJ, Currie PJ. New material of the large-bodied caenagnathid *Caenagnathus collinsi* from the Dinosaur Park Formation of Alberta, Canada. Cretac Res. 2015;54: 179–187. doi: 10.1016/j.cretres.2014.12.002

[43] Currie PJ. The first records of *Elmisaurus* (Saurischia, Theropoda) from North America. Can J Earth Sci. 1989;26: 1319–1324. doi: 10.1139/e89-111

[44] Sullivan RM, Jasinski SE. A new caenagnathid *Ojoraptorsaurus boerei*, N. Gen., N. Sp. (Dinosauria, Oviraptorosauria), from the Upper Cretaceous Ojo Alamo Formation (Naashoibito member), San Juan basin, New Mexico. Foss Rec. 2011;3: 418–428.

[45] Longrich NR, Barnes K, Clark S, Millar L. Caenagnathidae from the Upper Campanian Aguja Formation of West Texas, and a Revision of the Caenagnathinae. Bull Peabody Mus Nat Hist. 2013;54: 23–49. doi: 10.3374/014.054.0102

[46] Cullen TM, Simon DJ, Benner EKC, Evans DC. Morphology and osteohistology of a large-bodied caenagnathid (Theropoda, Oviraptorosauria) from the Hell Creek Formation (Montana): implications for size-based classifications and growth reconstruction in theropods. Pap Palaeontol. 2020; 1–17. 10.1002/spp2.1302.

[47] Funston GF, Currie PJ. A new caenagnathid (Dinosauria: Oviraptorosauria) from the Horseshoe Canyon Formation of Alberta, Canada, and a reevaluation of the relationships of Caenagnathidae. J Vertebr Paleontol. 2016;36: e1160910. doi: 10.1080/02724634.2016.1160910

[48] Ma W, Wang J, Pittman M, Tan Q, Tan L, Guo B, et al. Functional anatomy of a giant toothless mandible from a bird-like dinosaur: Gigantoraptor and the evolution of the oviraptorosaurian jaw. Sci Rep. 2017;7: 16247. doi: 10.1038/s41598-017-15709-7 29176627 PMC5701234

[49] Yu Y, Wang K, Chen S, Sullivan C, Wang S, Wang P, et al. A new caenagnathid dinosaur from the Upper Cretaceous Wangshi Group of Shandong, China, with comments on size variation among oviraptorosaurs. Sci Rep. 2018;8: 5030. doi: 10.1038/s41598-018-23252-2 29567954 PMC5864915

[50] Simon DJ, Varricchio DJ, Jin X, Robison SF. Microstructural overlap of Macroelongatoolithus eggs from Asia and North America expands the occurrence of colossal oviraptorosaurs. J Vertebr Paleontol. 2018;38: e1553046. doi: 10.1080/02724634.2018.1553046

[51] Simon DJ, Evans D. Osteohistology supports immature ontogenetic status of North American oviraptorosaurs *Apatoraptor pennatus* and *Chirostenotes pergracilis*. J Vertebr Paleontol Programs Abstr. 2021;41: 237–238.

[52] Simon DJ, Evans DC. Histological analysis of *Anzu wyliei* (Dinosauria, Oviraptorosauria) reveals variation in adult body size through time. J Vertebr Paleontol Programs Abstr. 2022;42: 306–307.

[53] Funston GF, Currie PJ, Tsogtbaatar C, Khishigjav T. A partial oviraptorosaur skeleton suggests low caenagnathid diversity in the Late Cretaceous Nemegt Formation of Mongolia. PLoS ONE. 2021;16: e0254564. doi: 10.1371/journal.pone.0254564 34252154 PMC8274908

[54] Lamm E-T. Preparation and Sectioning of Specimens. In: Padian K, Lamm E-T, editors. Bone histology of fossil tetrapods: advancing methods, analysis, and interpretation. Berkeley, CA: University of California Press; 2013. pp. 55–160. Available: https://california.universitypressscholarship.com/view/10.1525/california/9780520273528.001.0001/upso-9780520273528-chapter-4

[55] Schindelin J, Arganda-Carreras I, Frise E, Kaynig V, Longair M, Pietzsch T, et al. Fiji: an open-source platform for biological-image analysis. Nat Methods. 2012;9: 676–682. doi: 10.1038/nmeth.2019 22743772 PMC3855844

[56] Campione NE, Evans DC, Brown CM, Carrano MT. Body mass estimation in non-avian bipeds using a theoretical conversion to quadruped stylopodial proportions. Methods Ecol Evol. 2014;5: 913–923. doi: 10.1111/2041-210X.12226

[57] Marsh OC. Principal characters of American Jurassic dinosaurs. Am J Sci. 1878;Series 3 Vol. 16: 411–416. doi: 10.2475/ajs.s3-16.95.411

[58] Barsbold R. A new Late Cretaceous family of small theropods (Oviraptoridae n. fam.) in Mongolia. Dokl Akad Nauk SSSR. 1976;226: 221–223.

[59] Sternberg RM. A toothless bird from the Cretaceous of Alberta. J Paleontol. 1940;14: 81–85.

[60] Johnson KR, Nichols DJ, Hartman JH. Hell Creek Formation: a 2001 synthesis. The Hell Creek Formation and the Cretaceous-Tertiary Boundary in the Northern Great Plains: An Integrated Continental Record of the End of the Cretaceous. Geological Society of America; 2002. pp. 503–510.

[61] Funston GF, Mendonca SE, Currie PJ, Barsbold R. Oviraptorosaur anatomy, diversity and ecology in the Nemegt Basin. Palaeogeogr Palaeoclimatol Palaeoecol. 2018;494: 101–120. doi: 10.1016/j.palaeo.2017.10.023

[62] Currie P, Funston G, Osmolska H. New specimens of the crested theropod dinosaur *Elmisaurus rarus* from Mongolia. Acta Palaeontol Pol. 2016 [cited 9 Dec 2020]. doi: 10.4202/app.00130.2014

[63] Tsujimura K, Manabe M, Chiba Y, Tsuihiji T. Metatarsals of a large caenagnathid cf. *Anzu wyliei* (Theropoda: Oviraptorosauria) from the Hell Creek Formation in South Dakota, U.S.A. Can J Earth Sci. 2021 [cited 10 Apr 2021]. doi: 10.1139/cjes-2020-0171

[64] Osmólska H. Coossified tarsometatarsi in theropod dinosaurs and their bearing on the problem of bird origins. Palaeontol Pol. 1981;42: 79–95.

[65] Hutchinson JR. The evolution of hindlimb tendons and muscles on the line to crown-group birds. Comp Biochem Physiol A Mol Integr Physiol. 2002;133: 1051–1086. doi: 10.1016/s1095-6433(02)00158-7 12485692

[66] Carrano MT, Hutchinson JR. Pelvic and hindlimb musculature of *Tyrannosaurus rex* (Dinosauria: Theropoda). J Morphol. 2002;253: 207–228. 12125061 10.1002/jmor.10018

[67] Enlow DH. Principles of Bone Remodeling: An Account of Post-Natal Growth and Remodeling Processes in Long Bones and the Mandible. Thomas; 1963.

[68] Heck CT, Varricchio DJ, Gaudin TJ, Woodward HN, Horner JR. Ontogenetic changes in the long bone microstructure in the nine-banded armadillo (Dasypus novemcinctus). PLOS ONE. 2019;14: e0215655. doi: 10.1371/journal.pone.0215655 31022247 PMC6483220

[69] Curtin AJ, Macdowell AA, Schaible EG, Roth VL. Noninvasive histological comparison of bone growth patterns among fossil and extant neonatal elephantids using synchrotron radiation X-ray microtomography. J Vertebr Paleontol. 2012;32: 939–955. doi: 10.1080/02724634.2012.672388

[70] Curry Rogers K, Whitney M, D’Emic M, Bagley B. Precocity in a tiny titanosaur from the Cretaceous of Madagascar. Science. 2016;352: 450–453. doi: 10.1126/science.aaf1509 27102482

[71] Jasmina Hugi, Marcelo R. Sánchez-Villagra. Life History and Skeletal Adaptations in the Galapagos Marine Iguana (Amblyrhynchus cristatus) as Reconstructed with Bone Histological Data—A Comparative Study of Iguanines. J Herpetol. 2012;46: 312–324. doi: 10.1670/11-071

[72] Nacarino-Meneses C, Köhler M. Limb bone histology records birth in mammals. PLOS ONE. 2018;13: e0198511. doi: 10.1371/journal.pone.0198511 29924818 PMC6010216

[73] Funston GF, Currie PJ. A small caenagnathid tibia from the Horseshoe Canyon Formation (Maastrichtian): Implications for growth and lifestyle in oviraptorosaurs. Cretac Res. 2018;92: 220–230. doi: 10.1016/j.cretres.2018.08.020

[74] Funston GF, Wilkinson RD, Simon DJ, Leblanc AH, Wosik M, Currie PJ. Histology of Caenagnathid (Theropoda, Oviraptorosauria) dentaries and implications for development, ontogenetic edentulism, and taxonomy. Anat Rec. 2020;303: 918–934. doi: 10.1002/ar.24205 31270950

[75] Sander PM. Longbone histology of the Tendaguru sauropods: implications for growth and biology. Paleobiology. 2000;26: 466–488. doi: 10.1666/0094-8373(2000)026<0466:LHOTTS>2.0.CO;2

[76] Erickson GM, Curry Rogers K, Varricchio DJ, Norell MA, Xu X. Growth patterns in brooding dinosaurs reveals the timing of sexual maturity in non-avian dinosaurs and genesis of the avian condition. Biol Lett. 2007;3: 558–561. doi: 10.1098/rsbl.2007.0254 17638674 PMC2396186

[77] Lee AH, Werning S. Sexual maturity in growing dinosaurs does not fit reptilian growth models. Proc Natl Acad Sci. 2008;105: 582–587. doi: 10.1073/pnas.0708903105 18195356 PMC2206579

[78] Francillon‐Vieillot H, Buffrénil V de, Castanet J, Géraudie J, Meunier FJ, Sire JY, et al. Microstructure and Mineralization of Vertebrate Skeletal Tissues. Skeletal Biomineralization: Patterns, Processes and Evolutionary Trends. American Geophysical Union (AGU); 1990. pp. 175–234. doi: 10.1029/SC005p0175

[79] Woodward HN, Fowler EAF, Farlow JO, Horner JR. Maiasaura, a model organism for extinct vertebrate population biology: a large sample statistical assessment of growth dynamics and survivorship. Paleobiology. 2015;41: 503–527. doi: 10.1017/pab.2015.19

[80] Varricchio DJ. Late Cretaceous oviraptorosaur (Theropoda) dinosaurs from Montana. In: Tanke DH, Carpenter K, editors. Mesozoic Vertebrate Life. Bloomington: Indiana University Press; 2001. pp. 42–57.

[81] Bell PR, Currie PJ, Russell DA. Large caenagnathids (Dinosauria, Oviraptorosauria) from the uppermost Cretaceous of western Canada. Cretac Res. 2015;52: 101–107.

[82] Rhodes MM, Henderson DM, Currie PJ. Maniraptoran pelvic musculature highlights evolutionary patterns in theropod locomotion on the line to birds. Hedrick B, editor. PeerJ. 2021;9: e10855. doi: 10.7717/peerj.10855 33717681 PMC7937347

